# Views of Mexican outpatients with rheumatoid arthritis on sexual and reproductive health: A cross-sectional study

**DOI:** 10.1371/journal.pone.0245538

**Published:** 2021-01-28

**Authors:** Loraine Ledón-LLanes, Irazú Contreras-Yáñez, Guillermo Guaracha-Basáñez, Salvador Saúl Valverde-Hernández, Anayanci González-Marín, Ángel de Jesús Ballinas-Sánchez, Marta Durand, Virginia Pascual-Ramos

**Affiliations:** 1 Department of Biology of Reproduction, Instituto Nacional de Ciencias Médicas y Nutrición Salvador Zubirán, Mexico City, Mexico; 2 Department of Immunology and Rheumatology, Instituto Nacional de Ciencias Médicas y Nutrición Salvador Zubirán, Mexico City, Mexico; GPR Klinikum, GERMANY

## Abstract

**Background:**

Rheumatoid arthritis (RA) impacts sexual and reproductive health (SRH), which is a prominent component of a patient´s quality of life and highly influenced by the cultural background. The aim of the study was to explore the interest of Mexican outpatients with RA in SRH and to examine patient view on SRH.

**Methods:**

This cross-sectional study surveyed 303 consecutive outpatients with RA on their perceptions of SRH importance, SRH satisfaction, access to SRH information, preferences regarding SRH communication with healthcare professionals, and understanding of SRH (qualitative open-ended descriptions). Descriptive statistics and inferential analysis were used. Patient knowledge of each dimension of SRH was rated based on pre-specified criteria. Two assessors assigned ten major themes to each patient´s description of both dimensions of SRH.

**Results:**

Patients perceived their SRH as an important component of their general health and wished to address the topic, although few had access to such communication. Female patients assigned lesser importance to SRH, showed lesser degree of satisfaction with SRH, and expressed preference for a truthful physician. Age showed a linear association with individual survey responses, except for satisfaction with reproductive health dimension. There was a linear association between increased age and decreased years of formal education with a lower level of SRH knowledge. Ten major themes emerged for each of the two dimensions of the SRH construct, although most individual descriptions were assigned to one or two major themes.

**Conclusions:**

Further education and assessment of SRH in Mexican patients with RA is warranted.

## Introduction

In recent decades, the concept of reproductive health has evolved to offer a comprehensive and integrated approach to individual health needs related to sexuality and reproduction [1]. The Program of Action of the United Nations International Conference on Population and Development held in Cairo in 1994 [2] and the Fourth World Conference on Women sponsored by the United Nations held in Beijing in 1995 [3], adopted the following definition for reproductive health: “Reproductive health is a state of complete physical, mental, and social well-being, and not merely the absence of disease or infirmity, in all matters relating to reproductive system and to its functions and processes… It also includes sexual health, the purpose of which is the enhancement of life and personal relations, and not merely counselling and care related to reproduction and sexually transmitted diseases”. More recently, the World Health Organization (WHO) recognized that sexual health is an umbrella term that includes reproductive health [4]. In both definitions, a dissociation of the act of sex from reproduction is recognized, and sex is elevated from an act of reproductive instinct to an expression of love and confirmation of human bonding [1]. Accordingly, sexual and reproductive health (SRH) is a complex construct, integrated by two dimensions that need to be addressed separately. However, both SRH dimensions are important for quality of life at a personal level and for the individual’s integration into society [5]. Similar to health in general, SRH is promoted or undermined by lifestyle-related behaviors and simultaneously greatly influenced by the social, cultural, educational, and economic conditions of the community in which people are born and live [1]. Moreover, the relative importance of these determinants varies among countries, and their relevance varies for the two dimensions of the SRH construct, which limits the generalization of the results obtained from studies performed with populations with a different anthropologic framework.

Rheumatoid arthritis (RA) is a chronic inflammatory condition with worldwide distribution and female preponderance. The disease presents particular characteristics in the Latin American region, where it is differentiated by a younger age at presentation and extreme female preponderance compared to that in Caucasian populations [6–8]. RA can potentially impact every domain of a patient´s life [9], extending to SRH [10]. Two recent systematic literature reviews revealed an association between RA and sexual dysfunction among women and men [11,12]. Impaired sexual health has been attributed to RA-related symptoms and patient reduced function [13–22], the long and chronic course of the disease [23,24], associations with comorbid conditions, primarily psychological [22,25–27], the medications used [28,29], and hormone imbalance [30,31]. Importantly, chronic conditions, socio-demographic factors, and medications have also been considered risk factors associated with sexual dysfunction in the general population [32]. Meanwhile, most studies on reproductive health in rheumatic diseases have focused on fertility [33]. Reduced fertility has been described in women with RA by several studies [33–39], as a result of the disease process itself [35] or related to therapy [33], gonadal dysfunction [40], and due to medical advice and individual decisions [35], which are sometimes driven by fear of transmitting the disease to children or inability to care for small children [34]. In addition, lower parity [36] and subfertility [37–39] have also been associated with disease- and treatment-related variables in patients with RA.

Finally, RA is a prevalent disease among young Latin American women who are expected to be sexually active. SRH remains an area that patients and healthcare providers are reluctant to discuss in person [20,33,41]. Frequent reasons provided by healthcare professionals are poor training or education in sexual health, lack of relevant experience, religious or personal views, the belief that the topic is not important or appropriate, and embarrassment [42]. Josefsson et al. [14] confirmed that communication regarding sexual health between 63 Swedish patients with RA and their physicians was uncommon, whereas a considerable proportion of the patients expressed a desire for communication. In accordance, in a study with 74 RA patients from the UK, there was an 80% response rate (returned by pre-paid post) to a questionnaire concerning patient sexuality [20].

With the above considerations in mind, and given that SRH had strong cultural determinants, the aim of the study was to explore the interest of Mexican outpatients with RA in addressing SRH as part of their rheumatologic evaluation and to examine patient view on SRH, which has not been previously performed.

## Materials and methods

### Ethics

The study was performed in compliance with the Helsinki Declaration [43]. The Research Ethics Committee of the Instituto Nacional de Ciencias Médicas y Nutrición Salvador Zubirán (INCMyN-SZ) approved the study (reference number: IRE-1909). Written informed consent was waived due to the study characteristics (survey application). The Research Ethics Committee required anonymous application of the survey, due to sensitivity associated with the topic.

Consecutive patients from the outpatient clinic of the Department of Immunology and Rheumatology were invited to answer the survey, by trained personnel not involved in patient´s care, while waiting to receive their schedule consultation. Informed consent process was performed in all the patients who gave verbal consent and agreed to respond the survey. Verbal informed consent process was confirmed by the primary rheumatologist in charge of the patient, at the end of the consultation.

### Study design, study characteristics and target population

The study was cross-sectional and distributed a survey between January 20^th^ and February 29^th^ 2020 at the outpatient clinic of the Department of Immunology and Rheumatology of the INCMyN-SZ, a national referral and academic center for rheumatic diseases.

The survey was distributed to all the consecutive patients with RA (according to their primary rheumatologist criteria) who arrived at the outpatient clinic to attend a schedule visit (inclusion criteria). Exclusion criteria considered were additional (to RA diagnosis) rheumatic diagnosis, but secondary Sjögren syndrome, and patients who disagreed to participate.

During the study period, the outpatient clinic identified 436 patients who met inclusion criteria and were considered potential candidates to survey application, although 330 patients arrived to their schedule appointment. The characteristics of these patients, are summarized in S1 Table (Please refer to the S1 “Target population characteristics”, S1 Table). Briefly, patients were primarily middle-aged females, with substantial disease duration. Almost two-third of the patients had adequate control of the rheumatic disease according to the primary rheumatologist, meanwhile up to 46% of the patients referred joint pain, 24.4% morning stiffness and 9.8% substantial fatigue. There were 204 patients (61.8%) with at least one comorbid condition; among them, 84 (40.2%) had Systemic Arterial Hypertension, 70 (34.3%) type 2 Diabetes, 56 (27.4%) Hypothyroidism, 54 (26.5%) Dyslipidemia, 40 (19.5%) Osteoporosis and 10 patients (4.9%) had Depression. Finally, the majority of the patients were on disease modifying-anti-rheumatic drugs (DMARDs) and 28.2% on low doses of oral prednisone.

Finally, patients who completed the survey, corresponded to 91.8% of potential candidates and they were considered representative of the target population. Because of the anonymous application of the survey, their characteristics are not provided.

STROBE (Strengthening the Reporting of Observational Studies in Epidemiology) statement was followed [44] and the checklist of items that should be included is summarized in S2 Table (Please refer to the S2 “STROBE checklist for cross-sectional studies”, S2 Table).

### Survey development

Good practice in the conduct and reporting of survey research was followed [45].

The aim of the study was to explore the interest of Mexican outpatients with RA in addressing SRH as part of their rheumatologic evaluation and to examine patient view on SRH. A literature search failed to identify validated tools in Spanish, suitable to address the primary objective in our population.

Survey conceptualization was driven by clinical experience of a senior rheumatologist and of a PhD psychologist, previous clinical research in Mexican male patients with Systemic Lupus Erythematosus (SLE) from the same Institution, where patient’s interest in addressing the topic was identified [46], and the theoretical conceptualization of SRH [1]. The survey content was proposed by a committee consisting of one general practitioner, one senior rheumatologist in charge of an early RA clinic, and one PhD psychologist with experience in SRH. The committee agreed on five components to be included in the survey and suggested a reduced number of items to be included in the survey, due to the limited education of the target population.

Subsequently, ten individual items/questions, the scale responses to the ten items, and their distribution into the five components of the survey, were constructed by one coauthor. They were independently reviewed by the remaining two members of the committee who suggested splitting SRH in to sexual health and reproductive health, which were presented into one single item (N° 10). For this particular item that was intended to recall patient view of SRH, the expert committee considered it was convenient to direct patients to recognize both dimensions of the SRH construct. In addition, four demographic variables were included to be optionally filled by the patients: age, sex, level of formal education, and civil status (available in the last 130 surveys). The first version of the survey (SV_1_) was integrated and included 11 items. (Please refer to the S3 “Survey components, items and scale´s response”, S3 Table).

### Survey validation

Judgment experts determined the face and content validity of SV_1_. The expert committee consisting of a rheumatologist, reproductive health biologist, social worker, gynecologist, and bioethicist, who were blinded to each other’s evaluation, scored the following characteristics on standardized formats: adequate wording and appropriate language and meaning (for the target population) regarding individual items and instructions, adequacy of the item´s scale response, and relevance and pertinence of individual items to the survey purpose were also explored. As a result, item 3 was modified, and additional options for the item´s scale response were included for items 1–5 and item 9. In all cases, at least 80% agreement among experts was deemed necessary to approve modifications, and SV_2_ was integrated.

### Pilot testing

Fifty consecutive outpatients with RA were interviewed by two coauthors to assess their perception of instruction clarity, item adequate wording and meaning, and scale responses; standardized formats were used. Patients agreed on instruction clarity (85%), item adequate wording and meaning (90%), and adequacy of the scale response (95%). In addition, patients were directed to identify items they considered should be deleted, and to suggest items they considered should be added. There were no suggestions from patients but a blank section for additional comments that was added, and survey version 3 (SV_3_) was integrated and was considered suitable for final application (Please refer to the S4 “The sexual and reproductive health survey”, Spanish and English versions, S1 Appendix).

### Survey application

**Fig 1:**
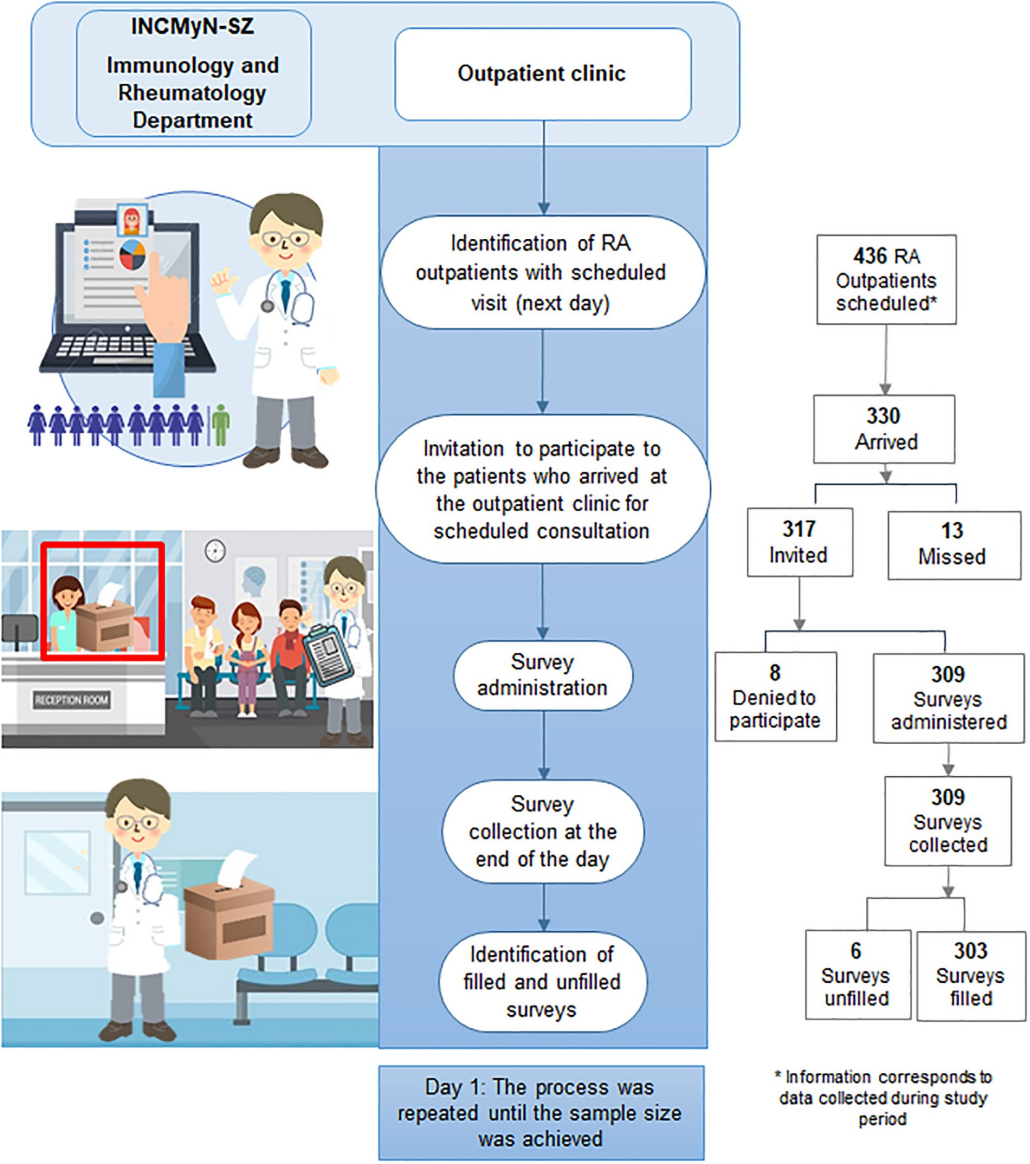
Survey administration steps.

First, all patients with a scheduled visit were identified on the day prior to their scheduled visit to the outpatient clinic of the Department of Immunology and Rheumatology. On the day of the scheduled visit, all patients who attended the outpatient clinic were invited to participate. Patients were instructed to read the survey, answer or not, and leave it at a designated area after their consultation. Every day, all surveys were collected at the end of the day, and answered surveys were identified. This procedure was repeated until the target sample of at least 248 completed surveys was achieved (Fig 1).

### Sample size calculation

The sample size for pilot testing was 50 patients, and the number of patients included followed recommendations for pilot testing [47]. For survey application, we estimated a sample size of at least 248 patients. We considered that 82% of the outpatients with RA would be interested in discussing SRH, based on local data obtained from men with SLE [46]. The outpatient clinic of the Department of Immunology and Rheumatology registered 1578 outpatients coded with RA during 2018 and a similar number was expected during 2019.

### Quantitative analysis

Descriptive statistics included frequencies and percentages, mean (± standard deviation [SD]) for normally distributed variables, and median (interquartile range [IQR]) for non-normally distributed variables. Descriptive statistics were also used to describe the distribution of major themes in the study population (see *Qualitative analysis* below). Face validity and content validity were examined by experts with agreement percentages.

Differences in the sex distribution of patient responses to the survey was compared with the X^2^ test.

Linear regression analysis was used to examine age as a predictor of response selection to individual items from the survey.

Kruskal–Wallis and X^2^ tests were used to compare the characteristics among patients classified according to their SRH knowledge (insufficient, borderline and sufficient knowledge).

Based on the distribution of the patients´ responses, scale responses were further grouped into five categories, instead of nine, as follows: (1) “Very Important/Important,” (2) “Somewhat important,” (3) “Of minor importance/Unimportant,” (4) “I don´t know/I haven´t thought about it,” and (5) “I don´t want to answer” for questions 1, 4, 5, and 9; (1)”Very satisfied/Satisfied,” (2)”Somewhat satisfied,” (3)”Mostly dissatisfied/Dissatisfied,” (4)”I don´t know/I haven´t thought about it,” and (5)”I don´t want to answer” for questions 2 and 3; and finally, (1)”Very frequent/Frequent,” (2)”Occasionally,” (3)”Rarely/Never,” (4)”I don´t know/I haven´t thought about it,” and (5)”I don´t want to answer” for questions 6 and 7.

A PhD psychologist rated patient knowledge of sexual health (item 10) and of reproductive health (item 11), according to the patient answers expressed in free-text, into three categories: “Insufficient knowledge,” “Borderline knowledge,” and “Sufficient knowledge” based on the WHO definition and pre-specified criteria (Please refer to the S5 “Criteria for sexual and reproductive health knowledge classification”, S4 Table).

Missing survey data (except for items 10 and 11) varied from 0% (for question 2) to 6.6% (for question 3). No imputation was performed.

All statistical analyses were performed using the Statistical Package for the Social Sciences version 21.0 (IBM Corp., Armonk, NY). A value of p < 0.05 was considered statistically significant.

### Qualitative analysis

Qualitative analysis was conducted on free-text comments related to patients understanding of SRH, using a brief thematic analysis approach [48].

First, three assessors read the free-text comments for item 10 (patient understanding of sexual health) and item 11 (patient understanding of reproductive health) and identified a list of categories for each dimension of the SRH construct, which was further organized into major themes. Then, the assessors met to discuss and agree on a list of ten major themes and their corresponding categories, which allowed an accurate description of the relevant characteristics of the content of each SRH dimension.

In the second step, two assessors independently assigned each patient´s answers regarding both dimensions of the SRH construct, to the categories and to the ten major themes from the list; when differences were present, they were resolved by consensus.

Finally, based on the content identified through the coding system of major themes and their corresponding categories, a brief qualitative analysis was carried out based on a general interpretation of the main subjective and social representations, which may support the expression of major themes related to their corresponding categories [48].

## Results

### Population characteristics

During the study period, there were 303 filled surveys (including two surveys with missing answers to item 8), and Fig 1 includes a flow chart that summarizes the recruitment process. The patients were primarily female (272 [89.8%]), with (median IQR) 53 (44–63) years of age. There were 68 (22.4%) patients with completed/incomplete elementary school education level, 80 (26.4%) patients who had graduated from middle school, 88 (29.0%) patients who had graduated from high school, and 67 patients (22.1%) who had completed a university degree. Finally, civil status-related data were available for 130 patients, half of whom (65 patients) were living with a partner.

### Survey results

**Fig 2:**
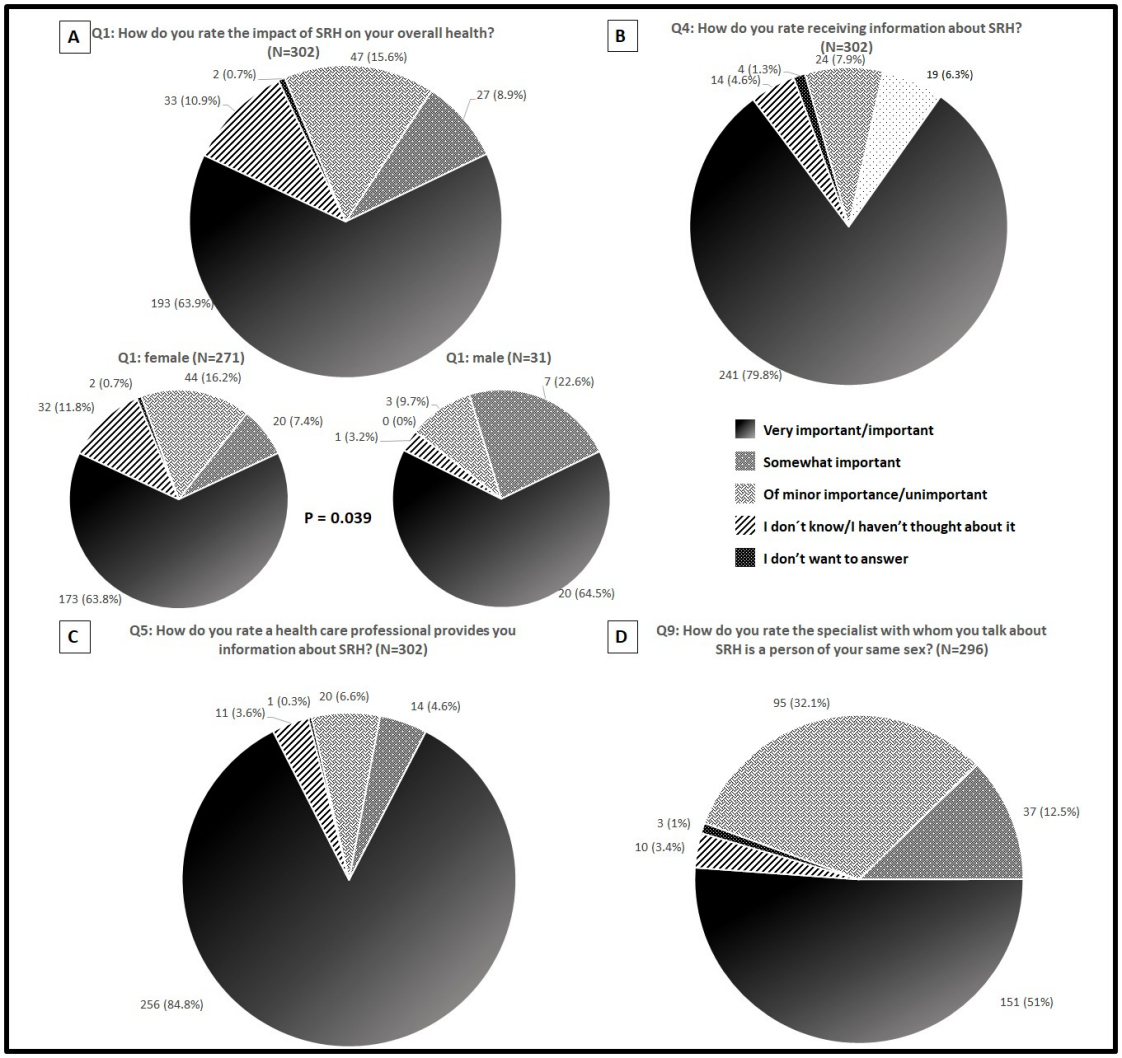
Patients responses distribution for questions 1 (A), 4 (B), 5 (C) and 9 (D).

**Fig 3:**
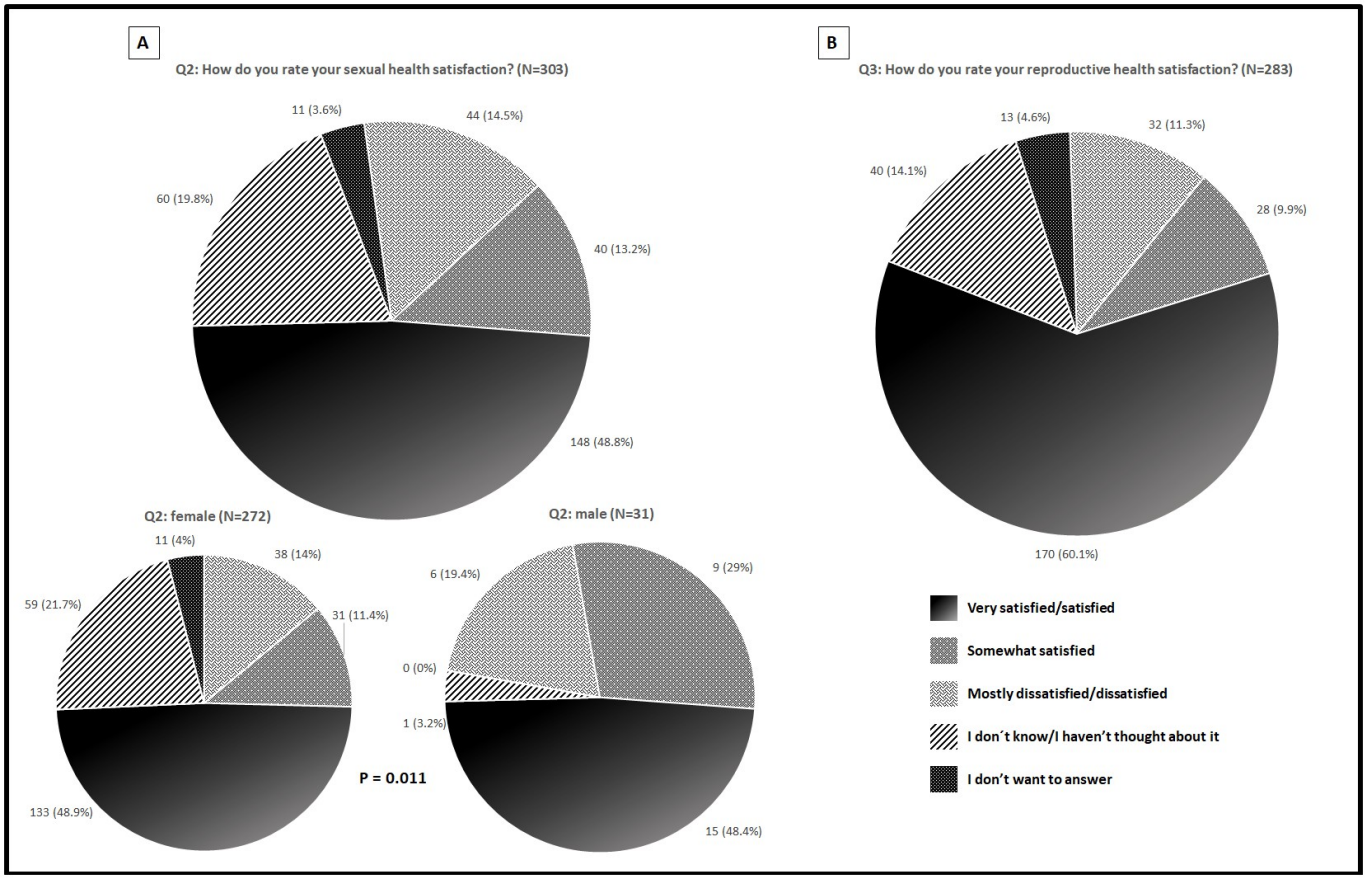
Patients responses distribution for questions 2 (A) and 3 (B).

**Fig 4:**
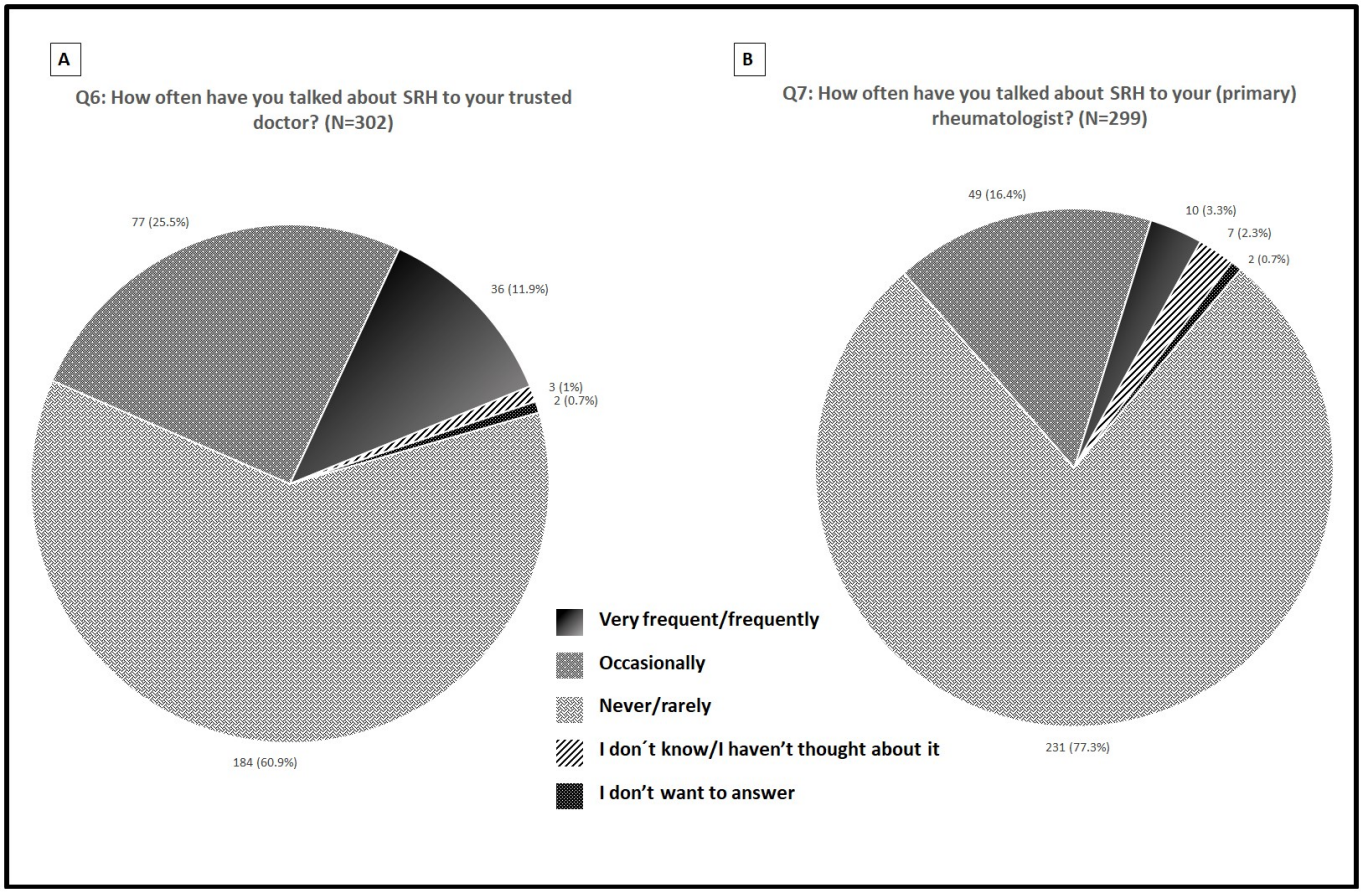
Patients responses distribution for questions 6 (A) and 7 (B).

Figs 2–4 summarize the distribution of patient responses to questions 1, 4, 5, and 9 (Fig 2 panels A, B, C and D, respectively), questions 2 and 3 (Fig 3 panels A and B, respectively), and questions 6 and 7 (Fig 4 panels A and B, respectively).

Overall, most patients rated questions 1 (63.9%), 4 (79.8%), 5 (84.8%) and 9 (51%) “Very important/Important.” Additionally, patients frequently rated questions 2 (48.8%) and 3 (60.1%) “Very satisfied/Satisfied”. Finally, most patients rated questions 6 (60.9%) and 7 (77.3%) as “Never/Rarely.”

The sex distribution of patient responses was then compared; there were differences for questions 1 and 2, as depicted in Figs 2(A) and 3(A), respectively. Regarding question 1, female patients selected more frequently the scale response “I don’t know/I haven´t thought about it” and “Of minor importance/Not important”; meanwhile, the opposite was true for the scale response “Somewhat important,” which was more frequently selected by male patients. Similarly, female patients selected more frequently response options “I don´t know/I haven´t thought about it” and “I don’t want to answer” for question 2, meanwhile, the opposite was true for the response options “Moderately satisfied” and “Mostly dissatisfied/Dissatisfied,” which were more frequently selected by male patients.

Two hundred and seventy female patients and 31 male patients answered question 8 (“Which healthcare provider, among those described below, would you like to talk with about SRH?”). The results of the patients’ responses are summarized in Table 1. Overall, most patients selected the “SRH expert” option (53.8%), without differences between male and female patients. More female patients selected a “trustful physician” as the second option (24.1% women vs. 9.7% men, p = 0.073); meanwhile, male patients selected the “Any physician” option more frequently than female patients did (8 [25.8%] vs. 19 [7%], p = 0.003). Finally, a similar proportion of male and female patients selected the “Rheumatologist” option.

**Table 1:** Patients responses distribution to question 8 (“Which healthcare provider, among those described below, would you like to talk with about SRH?”) and comparison between female and male patients responses.

	Overall population, N = 301*	Female patients, N = 270*	Male patients, N = 31*	p
SRH expert	162 (53.8)	148 (54.8)	14 (45.2)	0.345
Trustful physician	68 (22.6)	65 (24.1)	3 (9.7)	0.073
Rheumatologist	61 (20.3)	54 (20)	7 (22.6)	0.813
Any physician	27 (9)	19 (7)	8 (25.8)	0.003
Psychologist	22 (7.3)	22 (8.1)	0	0.145
None	19 (6.3)	17 (6.3)	2 (6.5)	1
Psychiatrist	6 (2)	6 (2.2)	0	1
Other health care provider	2 (0.7)	2 (0.7)	0	1
I don´t know	9 (3)	9 (3.3)	0	0.605
I don´t want to answer	2 (0.7)	2 (0.7)	0	1

*Data presented as N° (%).

### Association between age and individual responses

Age was examined as a predictor of response selection, for each individual item/question from the survey. Results are summarized in Table 2 and highlight that age predicted option response for every question except question 3 (“How do you rate your reproductive health satisfaction?”). Magnitudes of the relationship were similar across the questions but the direction differed, as negative correlations were found for questions 1–7, whereas a positive correlation was found for question 9.

**Table 2:** Linear regression analysis for age to predict response selection.

Questions	*β* coefficient	95% CI	*p* value
1. How do you rate the impact of SRH on your overall health?	-0.02	-0.03 to -0.01	≤0.001
2. How do you rate your sexual health satisfaction?	-0.02	-0.031 to -0.009	≤0.001
3. How do you rate your reproductive health satisfaction?	0.054	-0.006 to 0.015	0.411
4. How do you rate receiving information about SRH?	-0.024	-0.032 to -0.016	≤0.001
5. How do you rate a health care professional provides you information about SRH?	-0.012	-0.02 to -0.004	0.003
6. How often have you talked about SRH to your trusted doctor?	-0.013	-0.023 to -0.003	0.008
7. How often have you talked about SRH to your (primary) rheumatologist?	-0.017	-0.025 to -0.010	≤0.001
9. How do you rate the specialist with whom you talk about SRH is a person of your same sex?	0.014	0.002 to 0.026	0.020

CI = Confidence Interval. Question 8 not analyzed due to scale response.

### Patient knowledge of SRH

Overall, patients rated survey questions as easy to understand (75.4%), easy to answer (72.9%), and with adequate format (80.7%).

There were 71 (23.4%) and 72 (23.7%) missing data points, respectively, regarding the patient´s description of sexual health (item 10, open format) and of reproductive health (item 11, open format); among them, 58.7% coincided. There were 232 patient descriptions of sexual health dimension, and 20 (8.6%) were classified as “Insufficient knowledge,” 171 (73.7%) as “Borderline knowledge,” and 41 (17.7%) as “Sufficient knowledge” by the psychologist. Similarly, there were 231 patient descriptions of reproductive health dimension, and 16 (6.9%) were classified as “Insufficient knowledge,” 186 (80.5%) as “Borderline knowledge,” and 29 (12.6%) as “Sufficient knowledge.” We further explored differences among patients with insufficient, borderline, and sufficient knowledge regarding age, sex distribution, level of formal education, and civil status. The results are summarized in Table 3 for sexual health dimension and Table 4 for reproductive health dimension, and showed a linear association between increased age and decreased years of formal education with lower levels of knowledge for both dimensions of the SRH construct.

**Table 3:** Comparison of socio-demographic characteristics among patients classified based on their sexual health-related-knowledge level.

	Insufficient knowledge, N = 20	Borderline knowledge, N = 171	Sufficient knowledge, N = 41
(Median, Q25-Q75) age*	59.5 (51.3–65.5)	52 (42–62)	49 (51–58.5)
N° (%) of females	19 (95)	153 (89.5)	37 (90.2)
(Median, Q25-Q75) years of formal education*	7.5 (6–11.8)	11 (9–19)	12 (9–19)
N° (%) of patients living with partner^1^	7 (85.5)	31 (44.3)	7 (43.8)

^1^Restricted to 94 data available.

*p≤0.05 (Kruskall-Wallis and X^2^ tests).

**Table 4:** Comparison of socio-demographic characteristics among patients classified based on their reproductive health-related-knowledge level.

	Insufficient knowledge, N = 16	Borderline knowledge, N = 186	Sufficient knowledge, N = 29
(Median, Q25-Q75) age*	60 (58.3–63.3)	51 (42–61.3)	50 (34–59.5)
N° (%) of females	15 (93.8)	163 (87.6)	27 (93.1)
(Median, Q25-Q75) years of formal education*	6 (6–9)	11 (9–19)	12 (9–19)
N° (%) of patients living with a partner^1^	6 (85.7)	38 (50)	5 (41.7)

^1^Restricted to 95 data available.

*p≤0.05 (Kruskall-Wallis and X^2^ tests).

### Patient descriptions regarding sexual and reproductive health dimensions

There were 219 surveys (72.3%) with a description of sexual health (one description per survey), 73 surveys (24.1%) with missing information, and 11 surveys (3.6%) where patients stated that they could not provide a description. Among the former, the assessors first agreed on major theme assignment in 184 sexual health descriptions (84%) and reached a consensus on the remaining sexual health descriptions. Most of the sexual health descriptions (138 [63%]) were assigned to only one major theme, while the remaining descriptions (81 [37%]) were assigned to a combination of at least two major themes; in accordance, the median (IQR) of major themes/sexual health description was 1 (1–2). Similarly, there were 223 surveys (73.6%) with a description of reproductive health (one description per survey), 73 surveys (24.1%) with missing information, and seven surveys (2.3%) where patients stated that they could not provide a description. Among the former, the assessors first agreed on major theme assignment in 186 reproductive health descriptions (83.4%) and reached a consensus on the remaining descriptions. There were 92 (41.3%) reproductive health descriptions with only one major theme assigned, while the remaining descriptions (131 [58.7%]) were assigned to a combination of at least two major themes: the median (IQR) of major themes/reproductive health description was 1 (1–2).

### Description of major themes for each SRH construct

For each SRH construct, assessors agreed on the same 10 major themes: 1.- Information/knowledge; 2.- Overall prevention-oriented patient care; 3.- Family planning; 4.- Overall health; 5.- Well-being; 6.- Relationships and couple relationships; 7.- Biological aspects (primarily); 8.- Psychological aspects (primarily); 9.- Sexuality expression, and 10.- Holistic concept. A brief description of the main categories included in each major theme is provided in Table 5. Tables 6 (Sexual health) and 7 (Reproductive health) present illustrative quotes for main categories and each major theme identified in free-text comments.

**Table 5:** Major themes description for each dimension of SRH construct.

	Description of main categories	
	Sexual health dimension	Reproductive health dimension
Information/knowledge	11 categories	8 categories
	Being knowledgeable or informed regarding sexuality and sexual health, reproduction and reproductive health, family planning, and overall health.	Being knowledgeable or informed regarding reproduction, family planning, and pregnancy.
Overall prevention-oriented patient care	18 categories	14 categories
	(Self) care related to overall heath and sexuality, prevention of sexually transmitted infections and sexual diseases, and prevention of infections and diseases (in general).	General (self) care related to reproduction, receiving systematic prevention-oriented care related to the reproductive system organs and function, and healthcare related to pregnancy.
Family planning	5 categories	14 categories
	Contraception and contraceptive methods used, family planning issues, and unwanted pregnancy prevention.	Family planning issues (for example, making decision process about if, when and how to reproduce, planning the extension of the family), and reproductive desires.
Overall health	16 categories	11 categories
	Sexuality-related health and healthy sexual organs, the state of health need to optimal sexuality, overall health, and disease, comorbidities and age-related changes.	The (optimal) state of health for achieving reproduction, healthy reproductive organs, system and function, having a good overall health, and gynecological, obstetric and perinatal health.
Wellbeing	9 categories	6 categories
	Sexuality-related wellbeing, and overall wellbeing, both covering biological, psychological and social dimensions.	Well-being reproduction-related, and overall well-being covering biological, psychological and social dimensions.
Relationships and couple relationships	14 categories	5 categories
	Sexuality in couple, the values that fundament the couple relationship (for example, commitment, faithfulness, communication), and sexual satisfaction in couple.	The couple (intimate) relationship as the basis for reproduction, and talking about reproduction.
Biological aspects (primarily)	7 categories	9 categories
	Sexual organs and its functioning, as part or function body-related, and reproductive organs and its functioning.	Conceiving or having children, fertility, the life stage associated to biological changes related to reproduction, and pregnancy.
Psychological aspects (primarily)	5 categories	7 categories
	Attitudes regarding sexuality (for example: responsibility, autonomy, ethics, freedom), and reference to different expressions of identity (gender-related, sex-related, and sexual orientation-related).	Attitudes regarding reproduction (for example: responsibility, autonomy, awareness the reproductive decision-making process), reference to gender identity (female or male), and the assessment of reproductive health relevance.
Sexuality expression	12 categories	3 categories
	Optimal expression of sexuality, enjoying intercourse and sexual satisfaction, sexual activity and/or intercourse and their well-functioning, and references to sexual discomfort, disorders, or changes.	Sexual activity and/or intercourse and well-functioning of sexual activity.
Holistic concept	2 categories	1 category
	Balanced integration of physical, psychological, and social aspects in general and related to sexuality in particular.	Balanced integration of physical, psychological, and social aspects related to reproduction.

**Table 6:** Illustrative quotes (English and Spanish) for the main categories from each major theme identified in respondents’ description of sexual health.

Major themes	Main categories	Illustrative quote
**Information/knowledge**	Having information or knowledge about sexuality and sexual health	*"It is very important to have knowledge about sexual life so that it can be satisfactory”*.^1^
		*“Es muy importante tener conocimiento de la vida sexual para que pueda ser satisfactoria”*.
	Having information or knowledge about reproduction and reproductive health	*"Any type of information that we may have about our reproductive organs”*.^1^
		*“Cualquier tipo de información que podamos tener de nuestros órganos reproductores*.*”*
	Having information or knowledge about family planning	*"(*…*) to know what contraceptive methods exist so they are used in a correct way (*…*)"*.^1^
		*“(…) conocer y saber qué métodos anticonceptivos existen para utilizarlos de manera correcta (…)”*.
	Having information or knowledge about overall health	*"To be aware of how to be well"*.^1^
		*“Estar enterada de como estar bien”*.
**Overall patient care prevention-oriented**	(Self) care related to the overall health	*"Related to the means of prevention and care"*.^2^
		*“Relacionado a los medios de prevención y cuidados”*.
	(Self) care related to sexuality	*"(*…*) maximal cleanness*, *take precautions in sexual relationships"*.^2^
		*“(…) aseo al máximo*, *tener precauciones en la relaciones sexuales”*.
	Prevention of Sexually Transmitted Infections (STI) and sexual diseases	*"(*…*) healthy practices to avoid sexually transmitted diseases"*.^1^
		*“(…) prácticas sanas para evitar las enfermedades de transmisión sexual”*.
	Prevention of infections and diseases (in general)	*"Taking care in all aspects to prevent disease"*.^1^
		*“Cuidarse en todos los aspectos para prevenir enfermedad”*.
**Family planning**	Contraception and contraceptive methods’ use	*"To use family planning methods"*.^1^
		*“Utilizar métodos de planificación familiar”*.
	Family planning issues	*"Using (*…*) a contraceptive method so when you want to have a child he arrives at the planned time"*.^1^
		*“Es estar (…) con algún método anticonceptivo para que cuando desees tener un hijo llegue en el momento planeado”*.
	Unwanted pregnancies’ prevention	*“(…) For the prevention of unwanted pregnancies (…)”*.^1^
		*“(…) Para la prevención de embarazos no deseados (…)*.*”*
**Overall health**	Sexuality-related health and healthy sexual organs	*"Being healthy in the intimacy*, *without discharge or pain or secretions or odors"*.^1^
		*“Estar saludable en la intimidad*, *sin flujos ni dolores o secreciones ni olores”*.
	The state of health needed to optimal sexuality	*"I have to be healthy to have good sexual health"*. *(180)*
		*“Tengo que estar bien de salud para tener salud sexual bien”*.
	Overall health	*"To help to sustain a good state of health"*. *(281)*
		*“Ayudar a mantener un buen estado de salud”*.
	Disease, comorbidities and age-related changes	*"(*…*) other diseases that may interfere with the health left"*. *(286)*
		*“(…) otras enfermedades que puedan interferir en el resto de la salud”*.
**Well-being**	Sexuality-related wellbeing (covering biological, psychological and social dimensions)	*"It is the state of sexual health biopsychosocial well-being*, *in the presence or absence of any disease"*. *(16)*
		*“Es el estado de bienestar biopsicosocial de la salud sexual*, *en presencia o ausencia de alguna enfermedad”*.
	Overall wellbeing (covering biological, psychological and social dimensions)	*"For me it is part of being integrally well*, *in the mind and physical care (*…*)"*.*(130)*
		*“Para mí es parte de estar bien integralmente en los cuidados de la mente y físico (…)”*.
**Relationships and couple relationships**	Sexuality in couple	*"When you start having sex with your partner"*. *(91)*
		*“Cuando empiezas a tener relaciones sexuales con tu pareja”*.
	The values that fundament the couple relationship (for example: commitment, faithfulness, communication)	*"It is a way of healthy expressing our feelings within the couple*, *but if there is no understanding and respect it cannot be"*. *(248)*
		*“Es una forma de expresar sanamente nuestros sentimientos entre la pareja*, *pero si no hay comprensión y respeto no puede ser”*.
	Sexual satisfaction in couple	*"Having sex with your partner (…) and having it satisfying"*. *(86)*
		*“El tener relaciones sexuales con tu pareja (…) y que sea satisfactorio”*.
**Biological aspects**	Sexual organs and its functioning	*"Everything related to the genital apparatus regarding its functioning and state"*. *(246)*
		*“Todo lo relacionado con el aparato genital respecto a su funcionamiento y estado”*.
	A part or function body-related	*"I don’t know how to explain it but I think it is the function of the body that requires it so"*. (295)
		*“No sé cómo explicarlo pero pienso que es la función del cuerpo que lo requiere así”*.
	Reproductive organs and its functioning	*"Functioning of the reproductive organs (…)"*.^1^
		*“Funcionamiento de órganos reproductivos (…)”*.
**Psychological aspects**	Attitudes regarding sexuality (for example: responsibility, autonomy, ethic, freedom)	*"(…) To have full and conscious sexual relationships*, *when you choose and with whom you choose (…)*^1^
		*“(…) Tener relaciones sexuales plenas y conscientes*, *cuando tu elijas y con quien elijas (…)”*
	Reference to different expressions of identity (gender-related, sexual-related, and sexual orientation-related)	*"The way in which a person free from ties lives his sexual identity (…)"*.^2^
		*“La forma en que una persona libre de atadura vive su identidad sexual (…)”*.
**Sexuality expression**	The optimal expression of sexuality	*"Not having physical or mental impediment to have sexual relationships (…)"*.^1^
		*“No tener impedimento físico ni mental para tener relaciones sexuales (…)”*.
	Enjoying intercourse and sexuality satisfaction	*"To have sexual satisfaction in all ways*, *from a kiss to a satisfying sexual relationship"*.^1^
		*“Tener satisfacción sexual en todos los sentidos*, *desde un beso hasta una relación sexual satisfactoria”*.
	Sexual activity and/or intercourse and their well-functioning	*"(…) sexual practices to collaborate in a proper functioning"*.^1^
		*“(…) prácticas sexuales para colaborar en un funcionamiento adecuado”*.
	References to sexual discomfort, disorders or changes	*“(…) Any discomfort*, *(…) the changes that one presents with age and medications”*.^1^
		*“(…) cualquier molestia*, *(…) los cambios que uno va presentando con la edad y medicamentos”*.
**Holistic concept**	The balanced integration among physical, psychological and social aspects in general	*"Physical*, *mental and emotional issue of a person"*.^1^
		*”Cuestión física*, *mental y emocional de una persona”*.
	The balanced integration among physical, psychological and social aspects related to sexuality	*"The union of a mental*, *physical and social state related to sexuality and healthy relationships"*.^1^
		*“La unión de un estado mental*, *físico y social relacionado con la sexualidad y relaciones saludables”*.

^1^Female

^2^Male

**Table 7:** Illustrative quotes (English and Spanish) for the main categories from each major theme identified in respondents’ description of reproductive health.

Major themes	Main categories	Illustrative quote
**Information/knowledge**	Having information or knowledge about reproduction	*"To have knowledge about methods of reproduction*, *pregnancy and care"*.^1^
		*“Tener conocimientos para métodos de reproducción*, *embarazo y cuidados”*.
	Having information or knowledge about family planning	*"Learning about the methods of reproduction*, *planning and monitoring"*.^1^
		*“Conocer sobre los métodos de reproducción*, *planificación y seguimiento”*.
	Having information or knowledge about pregnancy	*"To know (…) how to take care of yourself when you want to get pregnant*, *and how to proceed in case of pregnancy"*. ^1^
		*“Saber (…) cómo cuidarse para cuando así se desee embarazarse*, *y cómo proceder en caso de embarazo”*.
**Overall patient care prevention-oriented**	General (self) care related to reproduction	*"Care and prevention*, *so that the reproduction would be satisfactory at any time"*.^1^
		*“Cuidados y prevención para que la reproducción sea satisfactoria en cualquier punto”*.
	Receiving systematic prevented-oriented care related to the reproductive system, organs and function	*"To follow a good control with your gynecologist regarding ovaries and womb"*.^1^
		*“Llevar un buen control con tu ginecólogo respecto a ovarios y matriz”*.
	Health care related to pregnancy	*"Care and attendance during pregnancy for avoiding problems with the baby"*.^1^
		*“Cuidados y atención durante el embarazo para evitar problemas con el bebé”*.
**Family planning**	Family planning issues (for example: the making decision process about if, when and how to reproduce, planning the extension of the family)	*"It is planning how many children you want to have (…)"*.^1^
		*“Es la planeación de cuantos hijos se quiere tener (…)”*.
	Reproductive desires	*“When you want to have a child (…)”*.^1^
		*“Cuando quieres tener un hijo (…)*.*”*
**Overall health**	The (optimal) state of health for achieving reproduction	*"To be in optimal physical and mental conditions if it is planned to conceive"*.^1^
		*“Estar en óptimas condiciones físicas y mentales si se planea concebir*.*”*
	Healthy reproductive’ organs, system and function	*“Optimal functioning for reproduction*, *healthy spermatozoids and eggs"*.^2^
		*“Funcionamiento óptimo para reproducirse*, *salud de espermatozoides y óvulos”*.
	Having a good overall health	*"Be in good health and be productive in the things I do"*.^1^
		*“Tener buen estado de salud y ser productivo en las cosas que hago”*.
	Gynecological, obstetric and perinatal health	*"Being able to have a pregnancy without any risk and without complication for the baby"*.^1^
		*“Poder tener un embarazo sin ningún riesgo y sin complicación para el bebé”*.
**Well-being**	Well-being reproduction-related	*"It implies physical well-being with the reproductive system"*.^1^
		*“Implica bienestar físico con el sistema reproductor”*.
	Overall well-being covering biological, psychological and social dimensions	*"It is a general state of physical*, *mental and social well-being*, *and absence of pain and disease"*.^1^
		*“Es un estado general de bienestar físico*, *mental y social*, *y ausencia de dolor y enfermedad*.*”*
**Relationships and couple relationships**	The couple relationship as the basis for reproduction	*"When it is wanted to have a child*, *the couple is important*.*"*^2^
		*“Cuando se quiere tener un hijo es importante la pareja*.*”*
	Talking about reproduction	*"Communication with the couple*, *knowing how many children for a better coexistence"*.^1^
		*“La comunicación con la pareja*, *saber cuántos hijos para una mejor convivencia”*.
**Biological aspects**	Conceiving or having children	*"To be able to conceive or to have a child or several"*.^1^
		*“Poder procrear o tener un hijo o varios”*.
	Fertility	*"Ability to be able to get pregnant without any risk"*.^1^
		*“Capacidad de poderse embarazar sin ningún riesgo”*.
	The life stage associated to biological changes related to reproduction	*"Stage in which your body begins to have hormonal changes and you become reproductive"*.^1^
		*“Etapa en la que tu cuerpo empieza a tener cambios hormonales y te vuelves reproductiva”*.
	Pregnancy	*"Everything related to (…) pregnancy"*.^1^
		*“Todo lo relacionado con (…) el embarazo”*.
**Psychological aspects**	Attitudes regarding reproduction (for example: responsibility, autonomy, to be aware about the reproductive making decision process)	*"To be prepared to conceive*, *to be aware*, *to do it with love*, *and not only for need it"*.^2^
		*“Prepararse para engendrar*, *estar consciente*, *hacerlo con amor*, *y no solo por necesidad”*.
	Reference to gender identity (female or male)	*"When the reproductive age’ woman has her family without any problem"*.^1^
		*“Cuando la mujer en edad reproductiva tiene a su familia sin ningún problema”*.
	Assessment of the reproductive health relevance	*"(…) It is important even to decide the children we want or not"*.^1^
		*“(…) Es importante incluso para decidir los hijos que deseamos o no”*.
**Sexuality expression**	Sexual activity and/or intercourse	*"Everything related to (…) the sexual act (…)"*.^1^
		*“Todo lo relacionado con (…) el acto sexual (…)”*.
	Sexual well-functioning	*“To have a good sexual relationship”*.^2^
		*“Tener una buena relación sexual”*.
**Holistic concept**	The balanced integration among physical, psychological and social aspects related to reproduction	*"The healthy way*, *mental and physical*, *with planning to create life"*.^1^
		*“La forma sana*, *mental y física con planeación para crear vida”*.

^1^Female

^2^Male

Finally, major themes distribution among the 219 and 223 surveys where the patients provided a description of sexual and of reproductive health, respectively, is summarized in Table 8. Overall prevention-oriented patient care, overall health and sexuality expression were the most frequently assigned themes to sexual health, while biological aspects, overall health, and overall prevention-oriented patient care were the most frequently assigned themes to reproductive health.

**Table 8:** Distribution of major themes in sexual health and reproductive health descriptions.

	N° (%) of sexual health descriptions with the major theme assigned	N° (%) of reproductive health descriptions with the major theme assigned
Information/knowledge	37 (16.9)	21 (9.4)
Overall prevention-oriented patient care	82 (37.4)	41 (18.4)
Family planning	12 (5.5)	30 (13.5)
Overall health	50 (22.8)	60 (26.9)
Well-being	30 (13.7)	16 (7.2)
Relationships and couple relationships	31 (14.2)	11 (4.9)
Biological aspects (primarily)	16 (7.3)	91 (40.8)
Psychological aspects (primarily)	17 (7.8)	34 (15.2)
Sexuality expression	49 (22.4)	7 (3.1)
Holistic concept	9 (4.1)	3 (1.3)

### Brief interpretation of patient descriptions of SRH

The same ten major themes were described for each patient definition of sexual health and of reproductive health, which could be considered an expression of the shared boundaries between the two dimension definitions from the patient perspective. These shared boundaries were specifically expressed in the sexual health definitions, which showed several major themes that included categories related to reproductive health (for example: references to reproduction, reproductive organs and their functioning, family planning issues, contraception, unwanted pregnancy prevention). Furthermore, the overlap between the major themes represented in the sexual health and reproductive health definitions could be considered either an expression of an integrative view from the patient perspective, or may be related to patient difficulty in effectively identifying both dimensions of the SRH construct. In general, the patients used more categories to define sexual health than reproductive health, except for the major themes “family planning,” “biological aspects (primarily),” and “psychological aspects (primarily)”. This could be considered an indicator of being more informed regarding sexual health as well as an indicator that the sexual health dimension elicits a greater diversity of meanings for the patient, compared with the reproductive health dimension.

Finally, it must be considered that in almost all major themes, patients combined the biomedical and psychosocial categories expressing a holistic perspective of sexual and reproductive health, framed in the context of overall health, which influences psychosocial (for example, relationships, values, communication, information, and satisfaction) and biomedical (for example, healthcare assistance, organs, systems and functions, and biological stages) domains to achieve a state of wellbeing.

## Discussion

The present study distributed an SRH-related-survey to Mexican outpatients with RA attending a tertiary care level center at the time of the study. The survey was locally developed; face and content validity were determined by an expert committee, and additionally confirmed with 50 patients who participated in a pilot test. The study revealed that Mexican patients with RA perceived their SRH as an important component of their general health and wished to address the topic with healthcare professionals, primarily with an SRH expert, although few had access to such communication. Meanwhile, most patients reported some degree of satisfaction with their SRH.

Nationality and ethnicity determine the particular cultural background that influences patient perceptions and views regarding the RA priority domains [49–52], trust in the physicians [53], and sexuality dimension and ways in which sexuality is conceptualized and explored [54]. Additionally, patients from our region expressed particular concerns and interests regarding their disease [55] and did not object to physician-centered/paternalistic patient-doctor relationships [56–59]. Ultimately, all conditions mentioned above integrate in a regional–cultural framework that shapes the results obtained herein. Accordingly, we consider that the study adds relevant information to the current knowledge on SRH in patients with RA, which has been conceived based on results from studies primarily performed in developed countries and with Caucasian populations [14,20,21,29,60,61].

To the best of our knowledge, only two studies have addressed this topic with Mexican patients [62,63], although one was limited to comparing obstetric prognosis before and after RA onset [62]. Skinner-Taylor et al. [63] described the SRH characteristics and contraceptive use of 135 Mexican women in childbearing age with a wide range of rheumatic diseases, among whom 35 had RA; authors found that 29% of their patients did not receive contraceptive counseling by any healthcare specialist. Lower rates of counseling regarding pregnancy and contraception have also been described in USA patients with rheumatic diseases [64–66]. Andreoli et al. [67] confirmed these results in 249 Italian patients with connective tissue diseases and 149 patients with chronic arthritis and additionally found that 34.5% of the patients with chronic arthritis, among whom 100 patients had RA, had never been asked by their rheumatologist regarding the desire to have children, while counseling was shown to play a role in increasing knowledge and had a positive impact on family planning.

We found that a significant number of the patients surveyed reported that they “Never/Rarely” talk about SRH with their rheumatologists; our approach to SRH was holistic, not limited to counseling for specific concerns, and targeted female and male patients of varying ages and with a wide range of disease durations. Similar percentages have been described for European patients with RA [14,20,68]. More recently, Twisttman et al. [69] found that up to 92% of 329 Danes RA patients, had never discussed sexual issues with a health care professional during the last 5 years.

Chakravarty et al. [70] conducted a multinational survey with 969 women aged 25–45 years with chronic inflammatory conditions from six countries (USA, UK, Germany, France, Italy, and Spain), 50 rheumatologists each from Germany, France, Italy, and the USA, and 100 gastroenterologists from the USA. The authors found that family planning and pregnancy were considered extremely important issues to be addressed by their patients and confirmed our results and those from other studies regarding patient-assigned importance to SRH [60,71]. Meanwhile, general practitioner (GPs)/primary care physicians and gynecologists were identified by the patients as frequently central to their discussions; it should be highlighted that in the UK, the patient’s GP and local midwife may oversee most of the pregnancy planning and management and could be considered surrogates of an “expert”. In addition, patients did also report that they wished to discuss family planning and pregnancy-related issues with their specialists (either rheumatologists or gastroenterologists). These results differed from those found in Danes RA patients were half of them stated that they did not want health care professionals to address sexual themes (occasionally) [69], and in UK RA patients with long-standing disease, where nurses were the patient´s preferred confidants to talk about sexual issues, followed by physicians [20]. In Swedish patients, the rheumatologist was the most frequent healthcare professional selected with whom to address sexual concerns, while French patients selected psychologists [68]. Our patients preferred an SRH expert and a trustful physician even more frequently than a rheumatologist [14].

The differences observed among populations regarding these preferences may be related to variation across countries in the healthcare system-related-working models, the (patient´s perception of the) training and communication skills of the healthcare providers involved in patient care, and the patient´s preferred communication pattern, which may be highly nuanced by the cultural background [42,53,72]. Importantly, high-power-distance cultural communities may assign higher value on healthcare professionals’ training and expertise. Finally, trust in physicians is a distinct and complex construct in itself [72], which has been associated with racial, cultural, socio-demographic, and disease-related variables [53,72,73] all of which may differ in our patients.

A second important finding was the differences between male and female patients in the answers provided to some components of the survey; female patients assigned lesser importance to SRH, showed lesser degree of satisfaction with SRH, and reported preference for a truthful physician to a greater extent than male patients.

Sexual problems have been reported more frequently by women either in RA populations [21,23,60,74] or in the general population [75–77]; meanwhile, the male sex has been associated with desire for sexual intercourse and frequency of sexual contact [74], which could be considered a surrogate for “importance assigned to SRH.” However, there is a hegemonic masculinity that requires confirmation and legitimization through active male involvement in sexuality [78]. In addition, men with RA reported a large impact on sexuality compared to women [21]. In a recent survey [68], French women with RA were reported to be more comfortable addressing sexual health with nurses and psychologists, while male patients selected rheumatologists or GPs; nurses and psychologists may represent trustful healthcare providers from the female perspective suggesting that women’s sexual concerns (extended to female patients with RA concerns) might be more psycho-affective than biologically driven [21] and should be addressed in an intimate environment.

The results discussed above highlight that there are culturally driven differences between men and women in the value and concerns assigned to sexuality, the differential impact of disease-related body damage on one´s body satisfaction and intimate relationships, and the social roles and expectations. Consequently, SRH-related conceptualizations and behaviors are expected to be different for women and men.

Third, age showed a linear association with responses to single items of the survey, except for satisfaction with reproductive health. The results could be aligned with the general conception that sexual activity decreases later in life, although it remains an important part of many relationships [79], and confirm results from previous studies where aging appeared to have an impact on the importance a patient places on sexual ability [20], although another study limited the (negative) association between age and sexual motivation/sexual activity and fantasies, to female patients [16]. Finally, the lack of association between age and satisfaction with reproductive health may be related to the population studied, primarily women in their 50s, who might have completed their family planning when the survey was conducted.

Fourth, in most patients, knowledge on SRH was rated as borderline; in addition, there was a linear association between increased age and decreased years of formal education with a lower level of knowledge. Finally, the qualitative analysis revealed ten major themes for each domain of the SRH construct, each with an integrative (biomedical, emotional, and social) approach, while most individual descriptions were limited to one, followed by two major themes.

When searching for additional information regarding SRH knowledge, studies with Mexican populations have targeted healthy adolescents and young individuals; in agreement with our results, the literature highlights a low to moderate level of general knowledge on sexuality and SRH [80], which might be partially explained by the limited perspective of the SRH-related topics taught in Mexican schools [80], in addition to biases integrated into national SRH programs [80]. Furthermore, advanced age might be an indicator of a subpopulation of patients with fewer years of education in our country; in fact, we found a low but significant inverse correlation between age and education level. SRH knowledge might be considered part of health literacy [81]. The relevance of achieving (health) literacy relies on literacy to impact health knowledge, health status, and access to health services, in addition to income level, occupation, education, housing, and access to medical care [81,82]; all of them are “red spots” in the Latin-American region and contribute to the current humanitarian crisis. Meanwhile, health literacy has been shown to positively impact different health-related outcomes [83,84], and should be integrated in to national education initiatives.

The first pre-specified criteria used to rate SRH knowledge adopted an integrative approach to SRH (biomedical, emotional, and social), which was recognized in the major themes from each dimension of the SRH construct emerging from the assigned categories. Meanwhile, most individual descriptions to each dimension were assigned to one or two major themes. As such, it appears that at the population level, SRH knowledge was more broadly conceptualized, as a reflection of the diversity of the patients included; meanwhile, at the individual level, SRH conceptualization was more limited, in accordance with SRH patient descriptions being primarily rate as reflective of borderline knowledge.

The widely varied content included in each definition of the SRH construct (ten major themes with a minimum of one to a maximum of 18 categories) might be consistent with the advantage of using open-ended questions to provide respondents with the freedom to express their opinion on the topic of research [85], which allows a better comprehension of the diversity of individual approaches. Moreover, and according to the major theme distribution, patients described each dimension of the SRH construct with a predominant content, they tended to define both dimensions through the major themes “overall prevented-oriented patient care” and “overall health”, indicating a strong representational association with health prevention and care, and with the health status as a preeminent condition for sexual health and reproductive health. The link between sexual health and reproductive health with different aspects of health and healthcare is probably related to the most widespread updated information on SRH, which is usually framed from a risk perspective, aimed to prevent sexually transmitted infections and unplanned pregnancies and is usually offered from healthcare services [80].

Patients’ definitions of sexual health included a group of categories related to the major theme “sexuality expression,” involving content related to sexual activity and intercourse, their well-functioning, sexual satisfaction and pleasure, and references to sexual disorders. It could express a representational separation between sexuality and reproduction, even when both appeared also to be mixed. The inclusion of the concept of sexual pleasure and satisfaction could indicate an investment from the human rights approach as well as the deeper significance of sexuality in patient lives and identities [86]. This appeared to be even more significant, considering that higher importance is attributed to reproduction than to sexuality, especially among Mexican women [78]. Nevertheless, it is quite possible that this approach (sexuality separated from reproduction) could coexist with the (inadequate) identification between sexuality and reproduction, which has been described in the Mexican population [78]. Finally, patient references to sexual disorders could be related to their personal experiences and to the previously described increased prevalence of sexual dysfunction in patients with RA [10–12,33]; it needs to be emphasized that RA patients from Latin America present distinct epidemiological characteristics (female preponderance and a younger age at presentation), which have the potential to shape personal SRH experience. Conversely, patient definitions of reproductive health included a group of categories related to the major theme “biological aspects (primarily),” especially represented by categories related to fertility, pregnancy, and having children. Considering that the study population primarily comprised female patients, this approach could be related to the preponderant role of motherhood in shaping female identity previously identified in Mexican women [78].

### Limitations

Some limitations of the study need to be addressed. First, the study was conducted at a single academic center located in a metropolitan area, and referred patients with RA may present unique characteristics. Additionally, the number of included male patients was limited, reflecting the worldwide female preponderance of RA; accordingly, the results might not be generalizable. Second, we used a locally developed survey, which lacked formal validation, although face and content validity were determined by an expert committee and confirmed in a pilot test with 50 outpatients with RA. Third, numerous factors may affect patient perceptions of SRH, and the survey content was certainly limited to assess all of them; in addition, some variables (marital status) were missing in a large number of patients. Fourth, the survey took place from January 20^th^ to February 29^th^ 2020 just a few weeks before the WHO declared the COVID-19 pandemic [87], which might have influenced the results. Finally, some patients with a scheduled appointment could not be invited to fill the survey, while few denied participation; their characteristics were not compared to those of the included patients, which could have biased results; nonetheless, the percentage of patients within each category was low (below 10%).

### Conclusions

Current knowledge regarding SRH in RA lacks the perspective of Latin American patients. Our study confirmed that SRH is a relevant outcome for Mexican outpatients with RA, although it is infrequently addressed by healthcare professionals. Age, sex, and literacy appear to shape Mexican patients’ SRH views and preferences for healthcare professionals with whom to address the topic; female Mexican patients valued trust. At the population level, Mexican patients with RA present a broader and integrative conceptualization (biomedical, emotional, and social) of each dimension of the SRH construct, framed from a preventive and assistant health approach. At the individual level, the integrative conceptualization remains, but it is more limited and translates into borderline SRH knowledge.

The study findings support the relevance of assessing patients with RA from a patient-centered approach, which might begin with the consideration that patients with RA could have SRH concerns. The patient-centered approach should be sensitive to the patient´s sex, life-course, and sociocultural particularities, to name the most relevant. There is need to provide patients with RA with SRH counseling, assistance, and rehabilitation interventions. It remains to be determined in our public health and academic environments, which healthcare professionals should be involved and how they might operationalize the provision of SRH care. A multidisciplinary approach should ideally be considered.

## Supporting information

S1 Table Target population characteristics. (PDF)

S2 Table STROBE checklist for cross-sectional studies. (PDF)

S3 Table Survey components, items and scale’s response. (PDF)

S4 Table Criteria for sexual and reproductive health knowledge classification. (PDF)

S1 Appendix The sexual and reproductive health survey. (PDF)

## References

[1] Fathalla MF, Fathalla MMF. Sexual and Reproductive Health: Overview In: Quah SR, Cockerham WC, editors. The International Encyclopedia of Public Health. Oxford Academic Press; 2017, pp. 481–490.

[2] United Nations. Report of the International Conference on Population and Development. A/94/10/18. 1995. [Cited 2020 Dec 01] https://www.un.org/en/development/desa/population/events/pdf/expert/27/SupportingDocuments/A_CONF.171_13_Rev.1.pdf.

[3] United Nations. Fourth World Conference on Women Platform for Action and the Beijing Declaration. 1995. [Cited 2020 Dec 01] https://www.un.org/en/events/pastevents/pdfs/Beijing_Declaration_and_Platform_for_Action.pdf.

[4] World Health Organization. Defining sexual Health. Report of technical consultation on sexual Health. WHO Publ [Internet]. 2002;(January):1–35. https://www.who.int/reproductivehealth/publications/sexual_health/defining_sexual_health.pdf.

[5] Pieber K, Stein KV, Herceg M, Rieder A, Fialka-Moser V, Dorner TE. Determinants of satisfaction with individual health in male and female patients with chronic low back pain. J Rehabil Med. 2012;44: 658–663. 10.2340/16501977-1010 22729793

[6] Pascual-Ramos V, Contreras-Yáñez I, Villa AR, Cabiedes J, Rull-Gabayet M. Medication persistence over 2 years of follow-up in a cohort of early rheumatoid arthritis patients: Associated factors and relationship with disease activity and with disability. Arthritis Res Ther. 2009;11: 1–11. 10.1186/ar2620 PMC2688260 19228421

[7] Cardiel MH, Pons-Estel BA, Sacnun MP, Wojdyla D, Saurit V, Marcos JC, et al Treatment of early rheumatoid arthritis in a multinational inception cohort of Latin American patients: The GLADAR experience. J Clin Rheumatol. 2012;18: 327–335. 10.1097/RHU.0b013e31826d6610 23047532

[8] Mody GM, Cardiel MH. Challenges in the management of rheumatoid arthritis in developing countries. Best Pract Res Clin Rheumatol. 2008;22: 621–641. 10.1016/j.berh.2008.04.003 18783741

[9] Geuskens GA, Burdorf A, Hazes JMW. Consequences of rheumatoid arthritis for performance of social roles—A literature review. J Rheumatol. 2007;34: 1639 17407220

[10] Zhang Q, Zhou C, Chen H, Zhao Q, Li L, Cui Y, et al Rheumatoid arthritis is associated with negatively variable impacts on domains of female sexual function: evidence from a systematic review and meta-analysis. Psychol Health Med. 2018 1 2;23: 114–125. 10.1080/13548506.2017.1338738 28635309

[11] Zhao S, Li E, Wang J, Luo L, Luo J, Zhao Z. Rheumatoid arthritis and risk of sexual dysfunction: A systematic review and metaanalysis. J Rheumatol. 2018;45: 1375–1382. 10.3899/jrheum.170956 29858239

[12] Perez-Garcia LF, te Winkel B, Carrizales JP, Bramer W, Vorstenbosch S, van Puijenbroek E, et al Sexual function and reproduction can be impaired in men with rheumatic diseases: A systematic review. Semin Arthritis Rheum. 2020;50: 557–573. 10.1016/j.semarthrit.2020.02.002 32165034

[13] Baldursson H, Brattström H. Sexual difficulties and total hip replacement in rheumatoid arthritis. Scand J Rheumatol. 1979;8: 214–216. 10.3109/03009747909114625 534315

[14] Josefsson KA, Gard G. Sexual Health in Patients with Rheumatoid Arthritis: Experiences, Needs and Communication with Health Care Professionals. Musculoskeletal Care. 2012;10: 76–89. 10.1002/msc.1002 22223288

[15] Shahar MA, Hussein H, Sidi H, Shah SA, Mohamed Said MS. Sexual dysfunction and its determinants in Malaysian women with rheumatoid arthritis. Int J Rheum Dis. 2012;15: 468–477. 10.1111/j.1756-185X.2012.01753.x 23083037

[16] van Berlo WTM, van de Wiel HBM, Taal E, Rasker JJ, Weijmar Schultz WCM, van Rijswijk MH. Sexual functioning of people with rheumatoid arthritis: A multicenter study. Clin Rheumatol. 2007;26: 30–38. 10.1007/s10067-006-0216-3 16508697

[17] Abdel-Nasser AM, Ali EI. Determinants of sexual disability and dissatisfaction in female patients with rheumatoid arthritis. Clin Rheumatol. 2006;25: 822–830. 10.1007/s10067-005-0175-0 16521053

[18] Saadat SH, Ramezani A, Ahmadi K. Sexual self-concept and general health in rheumatoid arthritis patients. Iran Red Crescent Med J. 2015;17: 0–3. 10.5812/ircmj.19005 26568849 PMC4640095

[19] Josefsson KA, Gard G. Women’s experiences of sexual health when living with Rheumatoid Arthritis—An explorative qualitative study. BMC Musculoskelet Disord. 2010;11: 240 10.1186/1471-2474-11-240 20950461 PMC2967510

[20] Hill J, Bird H, Thorpe R. Effects of rheumatoid arthritis on sexual activity and relationships. Rheumatology. 2003;42: 280–286. 10.1093/rheumatology/keg079 12595623

[21] Helland Y, Dagfinrud H, Kvien TK. Perceived influence of health status on sexual activity in RA patients: Associations with demographic and disease-related variables. Scand J Rheumatol. 2008;37: 194–199. 10.1080/03009740701867349 18465454

[22] El Miedany Y, El Gaafary M, El Aroussy N, Youssef S, Ahmed I. Sexual dysfunction in rheumatoid arthritis patients: Arthritis and beyond. Clin Rheumatol. 2012;31: 601–606. 10.1007/s10067-011-1891-2 22108779

[23] Karlsson B, Berglin E, Wållberg-Jonsson S. Life satisfaction in early rheumatoid arthritis: A prospective study. Scand J Occup Ther [Internet]. 2006;13: 193–199. 10.1080/11038120500462337 17042467

[24] Lin MC, Lu MC, Livneh H, Lai NS, Guo HR, Tsai TY. Factors associated with sexual dysfunction in Taiwanese females with rheumatoid arthritis. BMC Womens Health. 2017;17: 1–7. 28196535 10.1186/s12905-017-0363-5 PMC5309986

[25] Gutweniger S, Kopp M, Mur E, Günther V. Body image of women with rheumatoid arthritis. Clin Exp Rheumatol. 1999;17: 413–417. 10464550

[26] Yilmaz H, Polat HAD, Yilmaz SD, Erkin G, Kucuksen S, Salli A, et al Evaluation of Sexual Dysfunction in Women with Rheumatoid Arthritis: A Controlled Study. J Sex Med. 2012;9: 2664–2670. 10.1111/j.1743-6109.2012.02882.x 22906191

[27] Tristano AG. The impact of rheumatic diseases on sexual function. Rheumatol Int. 2009;29: 853–860. 10.1007/s00296-009-0850-6 19152092

[28] Hari A, Rostom S, Lahlou R, Bahiri R, Hajjaj-Hassouni N. Sexual function in Moroccan women with rheumatoid arthritis and its relationship with disease activity. Clin Rheumatol. 2015;34: 1047–1051. 10.1007/s10067-015-2888-z 25677567

[29] Tasiemski T, Angiaszwili-Biedna N, Wilski M. Assessment of objective and subjective quality of life in people with rheumatoid arthritis—preliminary study. Ortop Traumatol Rehabil. 2009;11: 346–359. 19828917

[30] Gerosa M, De Angelis V, Riboldi P, Meroni PL. Rheumatoid arthritis: A female challenge. Womens Health. 2008;4: 195–201. 10.2217/17455057.4.2.195 19072521

[31] Labrie F, Archer DF, Koltun W, Vachon A, Young D, Frenette L, et al Efficacy of intravaginal dehydroepiandrosterone (DHEA) on moderate to severe dyspareunia and vaginal dryness, symptoms of vulvovaginal atrophy, and of the genitourinary syndrome of menopause. Menopause. 2016;23: 243–256. 10.1097/GME.0000000000000571 26731686

[32] Lewis RW, Fugl-Meyer KS, Corona G, Hayes RD, Laumann EO, Moreira ED, et al Definitions/epidemiology/risk factors for sexual dysfunction. J Sex Med. 2010;7: 1598–1607. 10.1111/j.1743-6109.2010.01778.x 20388160

[33] Østensen M. Sexual and reproductive health in rheumatic disease. Nat Rev Rheumatol. 2017;13: 485–493. 10.1038/nrrheum.2017.102 28680137

[34] Clowse MEB, Chakravarty E, Costenbader KH, Chambers C, Michaud K. Effects of infertility, pregnancy loss, and patient concerns on family size of women with rheumatoid arthritis and systemic lupus erythematosus. Arthritis Care Res. 2012;64: 668–674. 10.1002/acr.21593 22344961

[35] Wallenius M, Skomsvoll JF, Irgens LM, Salvesen KÅ, Nordvåg BY, Koldingsnes W, et al Fertility in women with chronic inflammatory arthritides. Rheumatology (Oxford). 2011;50: 1162–1167. 10.1093/rheumatology/keq458 21292737

[36] Wallenius M, Skomsvoll JF, Irgens LM, Salvesen KÅ, Nordvåg BY, Koldingsnes W, et al Parity in patients with chronic inflammatory arthritides childless at time of diagnosis. Scand J Rheumatol [Internet]. 2012;41: 202–207. 10.3109/03009742.2011.641582 22360422

[37] Jawaheer D, Zhu JL, Nohr EA, Olsen J. Time to pregnancy among women with rheumatoid arthritis. Arthritis Rheum. 2011;63: 1517–1521. 10.1002/art.30327 21380995 PMC3106134

[38] Brouwer J, Hazes JMW, Laven JSE, Dolhain RJEM. Fertility in women with rheumatoid arthritis: Influence of disease activity and medication. Ann Rheum Dis. 2015;74: 1836–1841. 10.1136/annrheumdis-2014-205383 24833784

[39] Brouwer J, Fleurbaaij R, Hazes JM, Dolhain RJ, Laven JS. Subfertility in rheumatoid arthritis is often unexplained or caused by anovulation. Arthritis Care Res. 2017;69: 1142–1149. 10.1002/acr.23124 PMC5575464 27723275

[40] Henes M, Froeschlin J, Taran FA, Brucker S, Rall KK, Xenitidis T, et al Ovarian reserve alterations in premenopausal women with chronic inflammatory rheumatic diseases: Impact of rheumatoid arthritis, Behçet’s disease and spondyloarthritis on anti-Müllerian hormone levels. Rheumatol (United Kingdom). 2015;54: 1709–1712. 10.1093/rheumatology/kev124 25957439

[41] Irwin R. Sexual health promotion and nursing. J Adv Nurs. 1997;25: 170–177. 10.1046/j.1365-2648.1997.1997025170.x 9004026

[42] Donovan H, French K. Sexual and Reproductive Health RCN report on the impact of funding and service changes in England. Royal College of Nursing. 2018 [Cited 2020 Dec 01]. https://www.rcn.org.uk/professional-development/publications/pdf-006962.

[43] World Medical Association. Ethical principles for medical research involving human subjects. Eur J Emerg Med. 2001;8: 221–223. 10.1097/00063110-200109000-00010 11587468

[44] von Elm E, Altman DG, Egger M, Pocock SJ, Gøtzsche PC, Vandenbroucke JP. The Strengthening the Reporting of Observational Studies in Epidemiology (STROBE) statement: guidelines for reporting observational studies. Lancet. 2007;370: 1453–1457. 10.1016/S0140-6736(07)61602-X 18064739

[45] Kelley K, Clark B, Brown V, Sitzia J. Good practice in the conduct and reporting of survey research. Int J Qual Health Care. 2003;15: 261–266. 10.1093/intqhc/mzg031 12803354

[46] Merayo-Chalico J, Barrera-Vargas A, Morales-Padilla S, Reyna-De La Garza R, Vázquez-Rodríguez R, Campos-Guzmán J, et al Epidemiologic profile of erectile dysfunction in patients with systemic lupus erythematosus: The Latin American landscape. J Rheumatol. 2019;46: 397–404. 10.3899/jrheum.180292 30647184

[47] Steiner S, Norman G. Health Measurement Scales A practical guide to their development and use. Oxford University Press; 2003.

[48] Vaismoradi M, Turunen H, Bondas T. Content analysis and thematic analysis: implications for conducting a qualitative descriptive study. Nurs Health Sci. 2013;15: 398–405. 10.1111/nhs.12048 23480423

[49] Palominos PE, Gasparin AA, de Andrade NPB, Xavier RM, da Silva Chakr RM, Igansi F, et al Fears and beliefs of people living with rheumatoid arthritis: a systematic literature review. Adv Rheumatol. 2018;58: 1 10.1186/s42358-018-0001-4 30657055

[50] Graves H, Scott DL, Lempp H, Weinman J. Illness beliefs predict disability in rheumatoid arthritis. J Psychosom Res. 2009;67: 417–423. 10.1016/j.jpsychores.2009.01.006 19837204

[51] Kumar K, Gordon C, Toescu V, Buckley CD, Horne R, Nightingale PG, et al Beliefs about medicines in patients with rheumatoid arthritis and systemic lupus erythematosus: A comparison between patients of South Asian and White British origin. Rheumatology. 2008;47: 690–697. 10.1093/rheumatology/ken050 18375972

[52] Putrik P, Ramiro S, Hifinger M, Keszei AP, Hmamouchi I, Dougados M, et al In wealthier countries, patients perceive worse impact of the disease although they have lower objectively assessed disease activity: Results from the cross-sectional COMORA study. Ann Rheum Dis. 2016;75: 715–720. 10.1136/annrheumdis-2015-207738 26314921

[53] Berrios-Rivera JP, Street RL, Popa-Lisseanu MGG, Kallen MA, Richardson MN, Janssen NM, et al Trust in physicians and elements of the medical interaction in patients with rheumatoid arthritis and systemic lupus erythematosus. Arthritis Care Res. 2006;55: 385–393. 10.1002/art.21988 16739207

[54] Parker R. Sexuality, culture and society: Shifting paradigms in sexuality research. Cult Health Sex. 2009;11:251–266. 10.1080/13691050701606941 18608345

[55] Massone F, Martínez ME, Pascual-Ramos V, Quintana R, Stange L, Caballero-Uribe C V, et al Educational website incorporating rheumatoid arthritis patient needs for Latin American and Caribbean countries. Clin Rheumatol. 2017;36: 2789–2797. 10.1007/s10067-017-3866-4 29098475

[56] Pascual-Ramos V, Contreras-Yáñez I, Ruiz D, de la Luz Casas-Martínez M. Attitudes about principle of autonomy in Hispanic patients from a dynamic early rheumatoid arthritis cohort. Clin Exp Rheumatol. 2019;37: 608–614. 30620286

[57] Lazcano-Ponce E, Angeles-Llerenas A, Alvarez-Del Río A, Salazar-Martínez E, Allen B, Hernández-Avila M, et al Ethics and communication between physicians and their patients with cancer, HIV/AIDS, and rheumatoid arthritis in Mexico. Arch Med Res. 2004;35: 66–75. 10.1016/j.arcmed.2003.06.007 15036803

[58] Colmenares-Roa T, Huerta-Sil G, Infante-Castañeda C, Lino-Pérez L, Alvarez-Hernández E, Peláez-Ballestas I. Doctor-Patient Relationship between Individuals with Fibromyalgia and Rheumatologists in Public and Private Health Care in Mexico. Qual Health Res. 2016;26: 1674–1688. 10.1177/1049732315588742 27578852

[59] Thompson GA, Whiffen LH. Can Physicians Demonstrate High Quality Care Using Paternalistic Practices? A Case Study of Paternalism in Latino Physician–Patient Interactions. Qual Health Res. 2018;28: 1910–1922. 10.1177/1049732318783696 29962283

[60] Akkuş Y, Nakas D, Kalyoncu U. Factors affecting the sexual satisfaction of patients with rheumatoid arthritis and ankylosing spondylitis. Sex Disabil. 2010; 28: 223–232.

[61] Ahlmén M, Nordenskiöld U, Archenholtz B, Thyberg I, Rönnqvist R, Lindén L, et al Rheumatology outcomes: The patient’s perspective. A multicentre focus group interview study of Swedish rheumatoid arthritis patients. Rheumatology. 2005;44: 105–110. 10.1093/rheumatology/keh412 15381792

[62] Alvarez-Nemegyei J, Cervantes-Díaz MT, Avila-Zapata F, Marín-Ordóñez J. Desenlace obstétrico antes y después del inicio de la artritis reumatoide [Pregnancy outcomes before and after the onset of rheumatoid arthritis]. Rev Med Inst Mex Seguro Soc. 2011;49: 599–604. 22176821

[63] Skinner-Taylor CM, Perez-Barbosa L, Barriga-Maldonado ES, Cardenas-de la Garza JA, Diaz-Angulo JE, Figueroa-Parra G, et al Reproductive health counseling and contraceptive use in Mexican women with rheumatic diseases: a cross-sectional study. Rheumatol Int. 2020 8 14 10.1007/s00296-020-04679-1 32797280

[64] Birru Talabi M, Clowse MEB, Blalock SJ, Moreland L, Siripong N, Borrero S. Contraception Use Among Reproductive-Age Women With Rheumatic Diseases. Arthritis Care Res. 2019;71: 1132–1140. 10.1002/acr.23724 30106516 PMC6375807

[65] Ingram E, Claus L, Kolfenbach J, Wright G, Borgelt LM. Contraceptive Use in Women of Childbearing Ability With Rheumatoid Arthritis. J Clin Rheumatol. 2019 11 19 10.1097/RHU.0000000000001184 31789996

[66] Yazdany J, Trupin L, Kaiser R, Schmajuk G, Gillis JZ, Chakravarty E, et al Contraceptive counseling and use among women with systemic lupus erythematosus: A gap in health care quality? Arthritis Care Res. 2011;63: 358–365. 10.1002/acr.20402 21080446 PMC3115517

[67] Andreoli L, Lazzaroni MG, Carini C, Dall’Ara F, Nalli C, Reggia R, et al “Disease knowledge index” and perspectives on reproductive issues: A nationwide study on 398 women with autoimmune rheumatic diseases. Jt Bone Spine. 2019;86: 475–481. 10.1016/j.jbspin.2018.12.002 30579917

[68] Savel C, Cherillat MS, Berland P, Tronche AM, Soubrier M, Gerbaud L, et al French survey on the crossed needs on sexual health for chronic inflammatory rheumatism patients and healthcare professionals. Rheumatol Int. 2020;40: 1481–1491. 10.1007/s00296-020-04630-4 32621138

[69] Bay LT, Graugaard C, Nielsen DS, Möller S, Ellingsen T, Giraldi A. Sexual Health and Dysfunction in Patients With Rheumatoid Arthritis: A Cross-sectional Single-Center Study. Sexual Medicine. 2020;8: 615–630. 10.1016/j.esxm.2020.07.004 32912833 PMC7691882

[70] Chakravarty E, Clowse MEB, Pushparajah DS, Mertens S, Gordon C. Family planning and pregnancy issues for women with systemic inflammatory diseases: Patient and physician perspectives. BMJ Open. 2014;4:e004081 10.1136/bmjopen-2013-004081 24500612 PMC3918989

[71] Ryan S, Wylie E. An exploratory survey of the practice of rheumatology nurses addressing the sexuality of patients with rheumatoid arthritis. Musculoskeletal Care. 2005;3: 44–53. 10.1002/msc.25 17041993

[72] Freburger JK, Callahan LF, Currey SS, Anderson LA. Use of the Trust in Physician Scale in patients with rheumatic disease: Psychometric properties and correlates of trust in the rheumatologist. Arthritis Care Res. 2003;49: 51–58. 10.1002/art.10925 12579593

[73] Doescher MP, Saver BG, Franks P, Fiscella K. Racial and ethnic disparities in perceptions of physician style and trust. Arch Fam Med. 2000;9:1156–1163. 10.1001/archfami.9.10.1156 11115223

[74] Dorner TE, Berner C, Haider S, Grabovac I, Lamprecht T, Fenzl KH, et al Sexual health in patients with rheumatoid arthritis and the association between physical fitness and sexual function: a cross-sectional study. Rheumatol Int. 2018;38: 1103–1114. 10.1007/s00296-018-4023-3 29644435 PMC5953979

[75] Bortz WM, Wallace DH. Physical fitness, aging, and sexuality. West J Med. 1999;170: 167–169. 10214104 PMC1305535

[76] Heiman JR. Sexual dysfunction: Overview of prevalence, etiological factors, and treatments. J Sex Res. 2002;39: 73–78. 10.1080/00224490209552124 12476261

[77] Mercer CH, Fenton KA, Johnson AM, Wellings K, Macdowall W, McManus S, et al Sexual function problems and help seeking behaviour in Britain: National probability sample survey. Br Med J. 2003;327: 426–427. 10.1136/bmj.327.7412.426 12933730 PMC181259

[78] Galanti GA. The Hispanic family and male-female relationships. An overview. J Transcult Nurs. 2003;14:180–5. 10.1177/1043659603014003004 12861920

[79] Gott M, Hinchliff S. How important is sex in later life? The views of older people. Soc Sci Med. 2003;56: 1617–1628. 10.1016/s0277-9536(02)00180-6 12639579

[80] de Castro F, Rojas-Martínez R, Villalobos-Hernández A, Allen-Leigh B, Breverman-Bronstein A, Billings DL, et al Sexual and reproductive health outcomes are positively associated with comprehensive sexual education exposure in Mexican high-school students. PLoS One. 2018;13:e0193780 10.1371/journal.pone.0193780 29554152 PMC5858848

[81] NIH. Health Literacy Definition. Network of the National Library of Medicine web site. 2020. [Cited 2020 Dec 01] In NNLM web site [Internet]. https://nnlm.gov/initiatives/topics/health-literacy.

[82] Shiferaw KB, Tilahun BC, Endehabtu BF, Gullslett MK, Mengiste SA. E-health literacy and associated factors among chronic patients in a low-income country: a cross-sectional survey. BMC Med Inform Decis Mak. 2020;20: 181 10.1186/s12911-020-01202-1 32762745 PMC7407428

[83] Institute of Medicine (US) Committee on Health Literacy, Nielsen-Bohlman L, Panzer AM, Kindig DA, eds. Health Literacy: A Prescription to End Confusion. Washington (DC): National Academies Press (US); 2004 10.17226/10883 25009856

[84] Baker DW, Wolf MS, Feinglass J, Thompson JA, Gazmararian JA, Huang J. Health literacy and mortality among elderly persons. Arch Intern Med. 2007;167: 1503–1509. 10.1001/archinte.167.14.1503 17646604

[85] Dalati S, Marx Gómez J. Surveys and Questionnaires In: Modernizing the Academic Teaching and Research Environment. Germany: Springer International Publishing; 2018 pp. 175–186.

[86] Pizzarossa LB, Perehudoff K. Global Survey of National Constitutions: Mapping Constitutional Commitments to Sexual and Reproductive Health and Rights. Health Hum Rights. 2017;19: 279–293. 29302182 PMC5739376

[87] World Health Organization. Coronavirus disease 2019 (COVID-19): situation report, 51. World Health Organization; 2020. [Cited 2020 Dec 01] https://apps.who.int/iris/handle/10665/331475.

