# Supplementary material for: Views of Mexican outpatients with rheumatoid arthritis on sexual and reproductive health: A cross-sectional study
Source: PLoS One. 2021 Jan 28;16(1):e0245538. doi: 10.1371/journal.pone.0245538 (PMC7842945; doi:10.1371/journal.pone.0245538)
Supplement: S1 Table — (PDF) [file pone.0245538.s001.pdf]

Supplementary Table 1. Target population characteristics.

	**Target population**<br>**N=330**
**Socio-demographic characteristics**	
Females	299 (90.6)
Age, years, mean±SD	55.2 ±13.8
**Rheumatic disease characteristics**	
Disease duration, years, median (IQR)	9 (6-17)
Substantial disease activity level	91 (27.6)
Adequate control of the underlying disease	225 (68.2)
Joint pain (2 MD)	153 (46.4)
Morning stiffness (11 MD)	80 (24.4)
Substantial fatigue (41 MD)	32 (9.8)
**Comorbidities**	
≥1 comorbid condition	204 (61.8)
**Rheumatic disease-related treatment**	
DMARDs use	315 (95.5)
N˚ of DMARDs/patient, median (IQR)	2 (1-2)
Prednisone use	93 (28.2)

Data presented as N° (%) of patients unless otherwise indicated. IQR= Interquartile range.

SD=Standard Deviation. MD= Missing data. DMARDs= Disease modifying anti-rheumatic drugs.
