# Supplementary material for: Views of Mexican outpatients with rheumatoid arthritis on sexual and reproductive health: A cross-sectional study
Source: PLoS One. 2021 Jan 28;16(1):e0245538. doi: 10.1371/journal.pone.0245538 (PMC7842945; doi:10.1371/journal.pone.0245538)
Supplement: S3 Table — (PDF) [file pone.0245538.s003.pdf]

Supplementary Table 3. Survey components, items and scale´s response

Components	N° of items/questions	Scale response
Patient perception of SRH importance as part of their general health	1	Likert Scale (5 options) *Three additional options were added: "I do not know", "I haven´t thought about it" and "I do not want to answer"
Patient perceived SRH satisfaction	2	
Patient perceived access to SRH information	2	
Patient preferences about SRH communication with health professionals	4	
Patient understanding of SRH	2	Open answer

**Additional options were added for SV_2 to items 1-5 and item 9.*
