# Supplementary material for: Views of Mexican outpatients with rheumatoid arthritis on sexual and reproductive health: A cross-sectional study
Source: PLoS One. 2021 Jan 28;16(1):e0245538. doi: 10.1371/journal.pone.0245538 (PMC7842945; doi:10.1371/journal.pone.0245538)
Supplement: S4 Table — (PDF) [file pone.0245538.s004.pdf]

Supplementary Table 4. Criteria for sexual and reproductive health knowledge classification.

	Reproductive health	Sexual health
**Criterion 1**	Reference to a state of complete physical, mental and social well-being and not merely the absence of disease or infirmity, in all matters relating to the reproductive system and to its functions and processes.	Reference to a state of physical, emotional, mental and social well-being in relation to sexuality, and not merely the absence of disease, dysfunction or infirmity.
**Criterion 2**	Reference to the capability to reproduce and the freedom to decide if, when and how often to do so.	Reference that it requires a positive and respectful approach to sexuality and sexual relationships, as well as the possibility of pleasurable and safe sexual experiences, free of coercion, discrimination and violence.
**Criterion 3**	Reference to health-care services aimed at: Enabling safe pregnancy, childbirth and/or having a healthy infant/Counselling and giving access to safe, effective, affordable and acceptable methods of family planning and for fertility regulation/Infertility services/Access to safe elective abortion.	Reference to health-care services aimed at: Providing integral sexual education/Prevention of gender-based violence and providing assistance and support to victims/Prevention and control of HIV and sexually transmitted infections (STIs)/Providing psychosexual counselling and sexology care.
**Insufficient knowledge**	"I don't know" response or no response for criteria 1 to 3 is mentioned.	
**Borderline knowledge**	At least some content from one criterion is mentioned.	
**Sufficient knowledge**	Some content from at least two criteria is mentioned.	
