# Supplementary material for: Views of Mexican outpatients with rheumatoid arthritis on sexual and reproductive health: A cross-sectional study
Source: PLoS One. 2021 Jan 28;16(1):e0245538. doi: 10.1371/journal.pone.0245538 (PMC7842945; doi:10.1371/journal.pone.0245538)
Supplement: S1 Appendix — (PDF) [file pone.0245538.s005.pdf]

Encuesta sobre salud sexual y reproductiva para pacientes con artritis reumatoide de la consulta externa (Spanish version)

El Departamento de Reumatología e Inmunología del Instituto Nacional de Ciencias Médicas y de la Nutrición Salvador Zubirán (INCMNSZ), está interesado en conocer algunos aspectos sobre la salud sexual y reproductiva de las personas que reciben atención en este Servicio, así como sobre la comunicación que tienen sobre dicho tema con el personal de salud.

Por esta razón, solicitamos su ayuda contestando la siguiente encuesta. Su respuesta será anónima y confidencial. Los datos recogidos serán utilizados para mejorar la calidad de atención a nuestros pacientes.

Complete, por favor, los datos siguientes:

Edad en años: ______	Sexo:	Escolaridad:	Estado civil:
	☐ Mujer	☐ Sin estudios	☐ Soltero
	☐ Hombre	☐ Primaria	☐ Casado
		☐ Secundaria	☐ Viudo
		☐ Técnica	☐ Divorciado
		☐ Preparatoria	☐ Unión libre
Fecha: ______________________		☐ Superior	☐ Separado

A continuación, responda las siguientes preguntas marcando con una X la opción de respuesta que más se ajuste a su caso:

1. ¿Qué tan importante usted considera la salud sexual y reproductiva para su estado de salud en general? (seleccione solo una opción de respuesta)

- ☐ Muy importante
- ☐ Importante
- ☐ Moderadamente importante
- ☐ Poco importante
- ☐ Nada importante
- ☐ No sé
- ☐ No deseo contestar
- ☐ En este momento no lo he reflexionado

2. ¿Qué tan satisfecho o satisfecha se siente usted con su salud sexual? (seleccione solo una opción de respuesta)

- ☐ Muy satisfecho/a
- ☐ Satisfecho/a
- ☐ Moderadamente satisfecho/a
- ☐ Poco satisfecho/a
- ☐ Insatisfecho/a
- ☐ No sé
- ☐ No deseo contestar
- ☐ En este momento no lo he reflexionado

3. ¿Qué tan satisfecho o satisfecha se siente usted con su salud reproductiva? (seleccione solo una opción de respuesta)

- ☐ Muy satisfecho/a
- ☐ Satisfecho/a
- ☐ Moderadamente satisfecho/a
- ☐ Poco satisfecho/a
- ☐ Insatisfecho/a
- ☐ No sé
- ☐ No deseo contestar
- ☐ En este momento no lo he reflexionado

4. ¿Qué tan importante considera usted recibir información sobre la salud sexual y reproductiva? (seleccione solo una opción de respuesta)

- ☐ Muy importante
- ☐ Importante
- ☐ Moderadamente importante
- ☐ Poco importante
- ☐ Nada importante
- ☐ No sé
- ☐ No deseo contestar
- ☐ En este momento no lo he reflexionado

5. ¿Qué tan importante considera usted que el personal de salud que labora en el Instituto le proporcione información sobre salud sexual y reproductiva? (seleccione solo una opción de respuesta)

- ☐ Muy importante
- ☐ Importante
- ☐ Moderadamente importante
- ☐ Poco importante
- ☐ Nada importante
- ☐ No sé
- ☐ No deseo contestar
- ☐ En este momento no lo he reflexionado

6. ¿Qué tan frecuente usted ha hablado de su salud sexual y reproductiva con su médico/a de confianza? (seleccione solo una opción de respuesta)

- ☐ Muy frecuentemente
- ☐ Frecuentemente
- ☐ Ocasionalmente
- ☐ Raramente
- ☐ Nunca
- ☐ No sé
- ☐ No deseo contestar

En caso de haber contestado “Nunca” como opción de respuesta, ¿cuál fue el motivo?

____
____
____

7. ¿Qué tan frecuente usted ha hablado de su salud sexual y reproductiva con su reumatólogo/a tratante? (seleccione solo una opción de respuesta)

- ☐ Muy frecuentemente
- ☐ Frecuentemente
- ☐ Ocasionalmente
- ☐ Raramente
- ☐ Nunca
- ☐ No sé
- ☐ No deseo contestar

En caso de haber contestado “Nunca” como opción de respuesta, ¿cuál fue el motivo?

____
____
____

8. ¿Con qué especialista de salud preferiría conversar sobre su salud sexual y reproductiva? (puede seleccionar más de una opción de respuesta)

- ☐ Médico/a de confianza
- ☐ Cualquier médico/a
- ☐ Reumatólogo/a
- ☐ Psiquiatra
- ☐ Psicólogo/a
- ☐ Experto/a en el tema (sexólogo/a, ginecólogo/a, urólogo/a, andrólogo/a)
- ☐ Otro/a especialista ¿Cuál? ____
- ☐ Ninguno
- ☐ No sé
- ☐ No deseo contestar

9. ¿Qué tan importante considera usted que el/la especialista con quien usted converse sobre su salud sexual y reproductiva sea una persona de su mismo sexo? (seleccione solo una opción de respuesta)

- ☐ Muy importante
- ☐ Importante
- ☐ Moderadamente importante
- ☐ Poco importante
- ☐ Nada importante
- ☐ No sé
- ☐ No deseo contestar
- ☐ En este momento no lo he reflexionado

10. Explique a continuación, brevemente, qué usted entiende por salud sexual:

____
____
____
____

11. Explique a continuación, brevemente, qué usted entiende por salud reproductiva:

____
____
____
____

A continuación puede realizar cualquier observación o sugerencia que desee respecto a la presente encuesta:

____
____
____
____

Muchas gracias por su colaboración

The sexual and reproductive health survey (English version)

Please complete the following information

Age: ______ Sex: ☐ Female ☐ Male

Educational level: ☐ Without ☐ Elementary ☐ Middle school ☐ High school ☐ Vocational ☐ University

Marital status: ☐ Single ☐ Married ☐ Widowed ☐ Divorced ☐ Separated ☐ Living together

Date: ________________________

Then, answer the following questions by selecting the option that best suits your case

1. How do you rate the impact of sexual and reproductive health on your overall health? (Choose only one answer)

- ☐ Very important
- ☐ Important
- ☐ Somewhat important
- ☐ Of minor importance
- ☐ Unimportant
- ☐ I don't know
- ☐ I don't want to answer
- ☐ I haven't thought about it

2. How do you rate your sexual health satisfaction? (Choose only one answer)

- ☐ Very satisfied
- ☐ Satisfied
- ☐ Somewhat satisfied
- ☐ Mostly dissatisfied
- ☐ Dissatisfied
- ☐ I don't know
- ☐ I don't want to answer
- ☐ I haven't thought about it

3. How do you rate your reproductive health satisfaction? (Choose only one answer)

- ☐ Very satisfied
- ☐ Satisfied
- ☐ Somewhat satisfied
- ☐ Mostly dissatisfied
- ☐ Dissatisfied
- ☐ I don't know
- ☐ I don't want to answer
- ☐ I haven't thought about it

4. How do you rate receiving information about sexual and reproductive health? (Choose only one answer)

- ☐ Very important
- ☐ Important
- ☐ Somewhat important
- ☐ Of minor importance
- ☐ Unimportant
- ☐ I don't know
- ☐ I don't want to answer
- ☐ I haven't thought about it

5. How do you rate a health care professional provides you information about sexual and reproductive health? (Choose only one answer)

- ☐ Very important
- ☐ Important
- ☐ Somewhat important
- ☐ Of minor importance
- ☐ Unimportant
- ☐ I don't know
- ☐ I don't want to answer
- ☐ I haven't thought about it

6. How often have you talked about SRH to your trusted doctor? (Choose only one answer)

- ☐ Very frequent
- ☐ Frequent
- ☐ Occasionally
- ☐ Rarely
- ☐ Never
- ☐ I don't know
- ☐ I don't want to answer

In case that you answered "Never" as an option, what was the reason?

___

7. How often have you talked about SRH to your (primary) rheumatologist? (Choose only one answer)

- ☐ Very frequent
- ☐ Frequent
- ☐ Occasionally
- ☐ Rarely
- ☐ Never
- ☐ I don't know
- ☐ I don't want to answer

In case that you answered "Never" as an option, what was the reason?

___

8. Which healthcare provider, among those described below, would you like to talk with about sexual and reproductive health? (You can choose more than one option)

- ☐ Trustful physician
- ☐ Any physician
- ☐ Rheumatologist
- ☐ Psychiatrist
- ☐ Psychologist
- ☐ Sexual and reproductive health expert (sexologist, gynecologist, urologist, andrologist)
- ☐ Other health care provider ¿Which one? ____
- ☐ No one
- ☐ I don’t know
- ☐ I don’t want to answer

9. How do you rate the specialist with whom you talk about SRH is a person of your same sex? (Choose only one answer)

- ☐ Very important
- ☐ Important
- ☐ Somewhat important
- ☐ Of minor importance
- ☐ Unimportant
- ☐ I don’t know
- ☐ I don’t want to answer
- ☐ I haven’t thought about it

10. Please explain what you understand for sexual health

____
____
____
____

11. Please explain what you understand for reproductive health

____
____
____
____

Below you can make any observation or suggestion regarding this survey

____
____
____

Thank you very much for participating
