# Supplementary material for: Views of Mexican outpatients with rheumatoid arthritis on sexual and reproductive health: A cross-sectional study
Source: PLoS One. 2021 Jan 28;16(1):e0245538. doi: 10.1371/journal.pone.0245538 (PMC7842945; doi:10.1371/journal.pone.0245538)
Supplement: Attachment — Submitted filename: Response to reviewers.docx [file pone.0245538.s006.docx]

**RESPONSES TO EDITOR AND REVIEWERS**

**1. Please ensure that your manuscript meets PLOS ONE's style requirements, including those for file naming.**

*Response: We have revised PLOS ONE´s manuscript style requirements.*

**2. Please include additional information regarding the survey or questionnaire used in the study and ensure that you have provided sufficient details that others could replicate the analyses. For instance, if you developed a questionnaire as part of this study and it is not under a copyright more restrictive than CC-BY, please include a copy, in both the original language as well as the English version already provided, as Supporting Information.**

*Response: We have included in the S4-Appendix the Spanish version of the survey.*

**3. Please provide additional details regarding participant consent.**

**In the ethics statement in the Methods and online submission information, please ensure that you have specified (i) whether consent was informed and (ii) what type you obtained (for instance, written or verbal, and if verbal, how it was documented and witnessed). If your study included minors, state whether you obtained consent from parents or guardians. If the need for consent was waived by the ethics committee, please include this information.**

*Response: We have updated the ethics statement in Methods section and in the online submission.*

**4. In your Methods section, please provide additional information about the participant recruitment method and the demographic details of your participants.**

**Please ensure you have provided sufficient details to replicate the analyses such as:**

**a) a description of any inclusion/exclusion criteria that were applied to participant recruitment, b) a table of relevant demographic details, c) a statement as to whether your sample can be considered representative of a larger population.**

*Response: We have updated the methods section according to the editor´s recommendations. Data from the target population are presented in Supplementary Table 1.*

**Reviewer #1: Review of article: ”Views of Mexican outpatients with rheumatoid arthritis on sexual and reproductive health: a cross-sectional study”**

**Summary:**

**This study explores Mexican patients with RA and their interest in and views of sexual and reproductive health by using a cross-sectional design and a multi-methods approach in analyzing the data (descriptive and inferential statistics and quantifying themes and concepts for the open-ended questions). The study included 303 (272 women) patients with RA; civil status was retrieved from 130 participants of whom half were living with a partner.**

**The results showed a need from patients to discuss SRH issues, and that communication about SRH was not easy to access. Female gender, age, and education showed an association with specific areas in SRH. Ten major themes with two to eighteen categories for each of the two dimensions were constructed by the authors from the open-ended questions about the two dimensions, sexual health, and reproductive health, respectively. The authors conclude that there is a need for education and assessment of SRH in Mexican patients.**

**Comments:**

**Overall, the study addresses a sensitive and relevant issue in the care and treatment of patients with RA – specifically in the context of Mexican patients where studies seem to be absent on this, both sexual health and reproductive health.**

**The main limitations are the design of the study with a limited presentation of the qualitative part and the lack of representation of the patient group: patients with RA. It could be more visible that this is patients with a chronic illness, limited in many everyday activities.**

**There are some more specific considerations regarding methods and analyses that I would like to address:**

**1. One major limitation of this study is the lack of description of the cohort related to RA. It would strengthen the study to have a description of disability, levels of pain, fatigue, and disease activity. Rheumatology is well known for the use of parameters to measure these outputs and they are usually collected during consultations. Please reflect on why this was not considered and what it could have contributed to the study to use these.**

*Response: We appreciate RN, MHH Twisttmann comments. We did not provide the characteristics of the patients´ surveyed as we were required by Internal Review Board anonymous application (only four demographic variables were obtained in a limited number of the patients). In order to assess the limitation, we identified all the RA patients with a schedule consultation to the outpatient clinic during the study period, we reviewed their charts and obtained the following variables: Age, gender, years of disease duration, comorbid conditions, disease-related treatment, and disease activity, pain, morning stiffness and fatigue (present or absent). Additional variables were deemed relevant to describe the target population (like disability) but are not consistently recorded by the 21 rheumatologists in charge of the outpatient clinic. In the updated version of the manuscript, we are presenting this information in the “Study characteristics and target population” section and supplementary Table 1, as suggested by the editor and the reviewer. Finally, patients who completed the survey corresponded to 92% of the target population and we consider their characteristics to be similar to those from the target population.*

**2. You describe your method as a mixed-methods approach. I lack some information or theoretical considerations on this matter. Could it be, that the study is more a multi-methods study? Please comment on this. Mixed methods and multi-methods can be very relevant to illuminate different perspectives on the same subject. I do however find limitations in the qualitative part of the study, this I will address below, and could therefore suggest publishing the study as two different manuscripts. If you want to go through with the mixed/multi-methods approach, please explain what this design adds to your study and how you integrate the result – and how it may strengthen your discussion?**

*Response: We apologize for the misinterpretation driven by the term mixed-methods approach used in the first version of the manuscript. The study fits in quantitative research, although we propose to complement quantitative analysis (of the data from the survey) with qualitative analysis conducted on free-text comments related to patients understanding of SRH, using a brief thematic analysis approach. We have updated the manuscript and omitted from the text the term mixed-method.*

*We insist to include both analysis in the updated version as we consider they complement each other. This is the first study from our research group which addresses the topic (in RA patients). Our primary intention was to establish a “diagnosis” about (local) RA patients SRH perceptions and interest in addressing the topic. In such context, we considered relevant to additionally address patients view on SRH, to analyze them, and make them visible. We consider this approach will allow us to have a closer vision to our patients SRH vision, and to favor an effective and open communication.*

**3. Did you consider using questionnaires already developed on this issue (SRH)? What were the thoughts about developing a new questionnaire?**

*Response: There are some validated questionnaires that address the topic of SRH in RA patients, but as far as we know, no one has been culturally adapted, validated and intended to address our primary objective: To explore the interest of (Mexican) outpatients with RA in addressing SRH as part of their rheumatologic evaluation and to examine patient view on SRH. Jossefsson et al. (ref 14) developed a questionnaire and some of the items included, could have been used; nonetheless, the questionnaire was developed in Sweden and we could not find a validated Spanish version. Instead of translating, adapting to our culture and validating a pre-existing questionnaire, we opted to develop a tailored-survey, sensitive with the strong cultural determinants of SRH.*

**4. The conceptualization of the survey was developed by a committee. It is not clear how this processed – was it driven by experience alone, inspired by statements from patients, or driven by theory? Would the process have benefitted from including patient perspectives at this stage?**

*Response: We have updated the section and be more precise about survey conceptualization and development. Patients were included in the survey construction. During pilot testing, they were directed to identify items they considered should be deleted, and to suggest items they considered should be added (this have been updated in the corresponding section) in order to ensure that patients experience was captured. Certainly, patients could have been invited to participate since survey conceptualization. Whereas clinicians may be the best observers for certain RA aspects such as manifestations, only patients with the disease can report on more subjective elements. In our experience, this requires from local IRB to split the project in two, one for survey development and validation, and another one for survey application. By the time the project was conceived, COVID-19 pandemic was a menace for health care provision and we opted for a project we could develop without further delay.*

**How did you ensure that the components included the right or enough items?**

*Response: Patients were directed to identify items they considered should be deleted, and to suggest items they considered should be added (pilot testing) and the members of the expert committee were directed to evaluate the relevance and pertinence of individual items to the survey purpose (survey validation).*

**5. In the process of developing the questionnaires, did you consider questions related to rheumatoid arthritis? E.g. how the illness can affect SRH, as well as SRH can affect general health, and how disease management will affect SRH?**

*Response: The study was a first approach to RA patients and was intended to explore the interest of Mexican outpatients with RA in addressing SRH as part of their rheumatologic evaluation. We intentionally design a brief survey in order to achieve a high patient´s participation; at this point, we did not consider additional items. We are currently developing a new study, using validated questionnaires that addresses the relationship between the rheumatic disease and SRH.*

**6. Did you consider using a checklist in developing the questionnaire? It is not mentioned that you did, please comment on this. Likewise, did you use a checklist for the study (like CONSORT) as requested from the journal?**

*Response: Good practice in the conduct and reporting of survey research was followed and the reference has been added (currently number 45). In addition, STROBE statement (check list of items that should be included in reports from observational studies) was followed and the check-list has been added as Supplementary Table 2 (reference 44).*

**7. Could patient characteristics as mentioned above have benefited from supplemental information on e.g. BMI and co-morbidity like depression or osteoporosis? Several studies are addressing these factors and how they can limit sexual life and reproductive health.**

*Response: We have performed a chart review and included in the updated version of the manuscript some patient´s characteristics that describe overall target population. Although additional characteristics are important we consider they do not add much to the present study objective.*

**8. You describe sexual and reproductive health definitions in the introduction in a very fine way. I miss some information from the survey about distinguishing between these issues. Could you elaborate on this, please?**

*Response: RN, MHH Twisttmann is right. In the majority of the items, SRH was used as an umbrella term, which we consider could help patients to be comfortable with the topic (instead of facing a “test”) and complete the survey. Nonetheless, when addressing patient view/understanding of SRH (and when addressing satisfaction) we considered it was important to challenge patients to adopt a dissociated-dimension perspective of the SRH construct,* *so we could have a closer vision to our patients SRH vision.*

**9. The qualitative part of the study is lacking theoretical/methodological considerations. It is not quite transparent how the themes were constructed, how categories were chosen – and how and why they were similar/overlapping between the two dimensions. There is in my opinion, not a way that themes or categories emerge – they are constructed – and another approach for analyzing the text from patients could have provided more transparent data, analysis, interpretation, and conclusion. E.g. thematic analysis. Maybe this would have provided more disease-specific information/knowledge, that seems to be absent in the present material?**

**One way of transparency could also be to present the answers from the patients and how these text bites are transforming into the themes and categories. This is missing in the present study.**

*Response: We propose two additional tables that integrate illustrative quotes from patients and how they were transformed into main categories and major themes (Tables 6 and 7).*

**10. The discussion contains important and relevant considerations. However, some paragraphs could probably be shortened and more precise, e.g. the section on cultural and gender differences where there seems to be some overlap. Considering the sex differences, the authors may consider if the issues around body image/damage, social roles, etc., could have been addressed in some way in the survey? What consequences will this gender difference mean in practice, if any?**

*Response: We have shortened the discussion and be more precise. The issues proposed by RN, MHH Twisttmann are certainly relevant and need to be considered in future studies. In order to have a comprehensive approach to the relevance of these issues for patients SRH, we rather propose in depth interviews and/or semi-structured interviews (Study currently going on in our center).*

**In the discussion, age is mentioned as a factor related to the importance of sexual ability. You could maybe elaborate on this concerning the disease-specific factors as well – the debut age is different for women and men, so does this mean anything?**

*Response: As mentioned in the discussion, the study results are aligned with the general conception that sexual activity decreases later in life. The reviewer question is interesting and I had to go back to our local data from an early arthritis clinic, in order to provide an accurate answer. RA has some particular characteristics in Latin America, among them an extreme female prevalence (9 to 1) and a younger age at presentation (references are provided in the discussion section). Since 2004, I am in charge of the early arthritis cohort that was established for research purposes. We have data from 208 patients (followed-up for more than 12 years), among them 182 (87.5%) are female. The median (IQR) age at cohort entry in females was 37.7 years (27.1-48.5) vs. 41.3 (27.5-47.5) in male patients. There is no statistically significant difference between males and females age at cohort entry (p=0.546, Mann-Whitney U test). Nonetheless, I am not sure if the difference has any clinical significance for patients SRH, although I lean for a negative answer.*

**In the discussion section, patient knowledge or health literacy is addressed. I am unsure if the results from the present study show any difference from that of the general population. Could you please be more precise in this presentation?**

*Response: We agree with the reviewer, that our results are aligned with the general conception that education and health literacy are associated to (SRH) knowledge (similar to the discussion about age and sexual activity decrease). We consider the discussion is relevant because it makes visible the association and what is visible might be suitable for an (educational) intervention, in the appropriate direction. As state in the discussion section, national SRH programs offer a limited and biased perspective of SRH-related topics. We propose an updated paragraph.*

**In the discussion section, page 31 line 531, you state that “patients associate each dimension…”. From my point of view this was not patients associating this, but an interpretation from the authors during analyses of the qualitative/open-ended answers from the questionnaire?**

*Response: We totally agree and have updated the sentence.*

**The section page 32 is including patient statements more clearly and transparently – the study could benefit from more of these direct transparent transcripts of what the patients said/write.**

*Response: We have added tables 6 and 7 with illustrative quotes from patients, main categories and major themes.*

**The discussion could have benefitted from more perspectives on everyday life and sexual life in patients with RA – how are major topics like fatigue, pain disability, and psychological factors impacting how patients are perceiving SRH?**

*Response: We consider the updated discussion is tailored to the study objective, and includes relevant topics.*

**11. The limitations mentioned are all very relevant, very good considerations.**

*Response: Thank you for the comment.*

**12. References: There are some references in Spanish – please provide an English translation, or maybe choose an English reference when possible.**

*Response: We have updated references as suggested.*

**If you find it helpful, I have published a study on sexual health and dysfunction in patients with RA: “Sexual Health and Dysfunction in Patients With Rheumatoid Arthritis: A Cross-sectional Single-Center Study” https://pubmed.ncbi.nlm.nih.gov/32912833/**

*Response: Thank you for the reference. I really enjoy its reading, particularly the two-way-taboo concept. It has been included in the discussion.*

**Reviewer #2: The submitted manuscript is of fair interest to the field of rheumatoid arthritis, but I would still recommend major revision of the manuscript, as it is somewhat lacking in clinical relevance.**

**Major Criticism:**

**- It is unfortunate that the authors did not consider specific RA-related symptoms and patient reduced function, such as the long and chronic course of the disease, associations with comorbid conditions, the medications used, and hormone imbalances, which are known to affect social functioning in RA-Patients. The inclusion of this type of data would have made the manuscript very much more relevant. Is there any possibility to include data of the subjects on disease duration, number of medications used, number of co-morbidities, etc, to investigate associations between SRH and the before mentioned factors?**

*Response: We appreciate the comments. We did not provide the characteristics of the patients´ surveyed as we were required by Internal Review Board anonymous application (only four demographic variables were obtained in a limited number of the patients). In order to assess the limitation, we identified all the RA patients with a schedule consultation to the outpatient clinic during the study period, we reviewed their charts and obtained the following variables: Age, gender, years of disease duration, comorbid conditions, disease-related treatment, and disease activity, pain, morning stiffness and fatigue (present or absent). Additional variables were deemed relevant to describe the target population (like disability) but are not consistently recorded by the 21 rheumatologists in charge of the outpatient clinic. In the updated version of the manuscript, we are presenting this information in the “Study characteristics and target population” section and supplementary Table 1, as suggested by the editor and the reviewers.*

*Regarding the potential association between some aspects of the underlying rheumatic disease and SRH, although relevant, it does not correspond to the current study objective. We are developing a new study, using validated questionnaires that addresses the relationship between the rheumatic disease and SRH.*

**Minor Criticism**

**- Could the authors provide a table with baseline characteristics (Age, gender, socio-demographic factors)?**

*Response: Supplementary Table 1 includes the characteristics of the target population, defined as the potential candidates for survey application. Patients who finally completed the survey, corresponded to 91.8% of the potential candidates and they were considered representative of the target population. Because of the anonymous application of the survey, their characteristics cannot be provided.*

**- Did the authors consider using an already available questionnaire on sexual and reproductive health as to developing a new one themselves. Please elaborate.**

*Response: There are some validated questionnaires that address the topic of SRH in RA patients, but as far as we know, no one has been culturally adapted, validated and intended to address our primary objective: To explore the interest of (Mexican) outpatients with RA in addressing SRH as part of their rheumatologic evaluation and to examine patient view on SRH. Jossefsson et al (ref 14) developed a questionnaire and some of the items included, could have been used; nonetheless, the questionnaire was developed in Sweden and we could not find a validated Spanish version. Instead of translating, adapting to our culture and validating a pre-existing questionnaire, we opted to develop a tailored-survey, sensitive with the strong cultural determinants of SRH.*
